# Manipulation of the dually thermoresponsive behavior of peptide‐based vesicles through modification of collagen‐like peptide domains

**DOI:** 10.1002/btm2.10145

**Published:** 2019-10-16

**Authors:** Lucas C. Dunshee, Millicent O. Sullivan, Kristi L. Kiick

**Affiliations:** ^1^ Department of Chemical and Biomolecular Engineering University of Delaware Newark Delaware; ^2^ Department of Biomedical Engineering University of Delaware Newark Delaware; ^3^ Department of Materials Science and Engineering University of Delaware Newark Delaware

## Abstract

Materials that respond to temporally defined exogenous cues continue to be an active pursuit of research toward on‐demand nanoparticle drug delivery applications, and using one or more exogenous temperature stimuli could significantly expand the application of nanoparticle‐based drug delivery formulations under both hyperthermal and hypothermal conditions. Previously we have reported the development of a biocompatible and thermoresponsive elastin‐*b*‐collagen‐like polypeptide (ELP‐CLP) conjugate that is capable of self‐assembling into vesicles and encapsulating small molecule therapeutics that can be delivered at different rates via a single temperature stimulus. Herein we report the evaluation of multiple ELP‐CLP conjugates, demonstrating that the inverse transition temperature (*T*_t_) of the ELP‐CLPs can be manipulated by modifying the melting temperature (*T*_m_) of the CLP domain, and that the overall hydrophilicity of the ELP‐CLP conjugate also may alter the *T*_t_. Based on these design parameters, we demonstrate that the ELP‐CLP sequence (VPGFG)_6_‐(GPO)_7_GG can self‐assemble into stable vesicles at 25°C and dissociate at elevated temperatures by means of the unfolding of the CLP domain above its *T*_m_. We also demonstrate here for the first time the ability of this ELP‐CLP vesicle to dissociate via a hypothermic temperature stimulus by means of exploiting the inverse transition temperature (*T*_t_) phenomena found in ELPs. The development of design rules for manipulating the thermal properties of these bioconjugates will enable future modifications to either the ELP or CLP sequences to more finely tune the transitions of the conjugates for specific biomedical applications.

## Graphical abstract

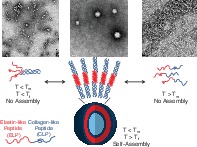

## 1 INTRODUCTION

Thermoresponsive materials have been of prevailing interest for the last decade and continue to be widely studied for purposes ranging from biomaterials engineering^1^ to the textiles industry.^2^ Specifically, externally‐triggered, stimuli‐responsive materials have been developed for long‐term application in coated surfaces for anti‐fouling films,^3^ cellular adhesion and removal devices,^4^ anti‐opsonization materials,^5^ and on‐demand drug and gene‐releasing nanoparticles.^6^, ^7^, ^8^, ^9^ Drug delivery applications would benefit in particular from the spatio‐temporal control that is provided by thermoresponsive materials since a locally altered temperature profile allows for site‐specific release of therapeutics and thereby is ideal to enable improved drug targeting.^10^ Thermoresponsive materials also would allow selection of when a drug is to be released (i.e., based upon when the external temperature stimulus is applied) relative to a patient's condition, and this temporal control would in turn allow the local concentration of the drug to be maintained above a therapeutically relevant threshold at any given time.^11^

Dual stimuli‐responsive polymeric nanoparticles have garnered particular research interest in recent years. These dual stimuli‐responsive nanoparticles often employ thermal responsivity as one of the two stimuli, with designs employing pH/temperature‐, magnetic/temperature‐, light/redox‐, to pH/redox‐responsive particles among others^6^, ^12^, ^13^, ^14^; moreover, the behavior of many dual‐responsive particles is tailored for a specific disease. For instance, Zhang et al. produced nanoparticles from diblock copolymers that were both pH and thermoresponsive, and they showed increased drug release in the presence of both a higher temperature (e.g., >20°C) and a lower pH (e.g., <6.8) stimuli that are both commonly found in cancerous tumor microenvironments.^6^ Similarly, Sun et al. reported the synthesis of pH and reduction sensitive polymersomes for the delivery of apoptotic proteins to cancer cells that typically possess an imbalance in reduction potential.^15^, ^16^ One general challenge in applying such approaches in a clinical setting is that the conditions in endogenous tumor environments are often difficult to predict and commonly vary from person to person.^17^

While the specificity offered by a mixed dual stimuli‐responsive (e.g., two stimuli that differ from one another in type) system is desired in certain cases, dual temperature‐responsive particles would have broad and diverse applications for drug delivery in multiple disease models. Specifically, a dual thermoresponsive particle able to dissociate upon application of either hyperthermal or hypothermal temperatures would be relevant for therapeutic regimens that include a co‐operative applied temperature stimulus. For instance, hyperthermal treatments have been used in conjunction with small molecule drugs for the treatment of cancer.^18^ Similarly, hypothermal therapies have been shown to aid the delivery of small molecules in diseases such as osteo‐ and rheumatoid arthritis,^19^ embolic stroke,^20^ and spinal cord injuries.^21^ In both cases, the same nanoparticle formulation could be used for treatment with only the drug cargo being altered for different diseases.

Additionally, dual thermoresponsive particles may offer unique drug release kinetics from each of the two different temperature‐responsive domains,^22^, ^23^ enable unique alterations in particle morphology to increase targeting ligand valency/presentation on the particle surface,^24^, ^25^ or enable nanoparticle aggregation^6^, ^26^ to site‐specifically trap particles and their cargo in specific locations.

Interestingly, there are very few reports that focus on dual thermoresponsive polymeric nanoparticle systems.^27^, ^28^, ^29^ Of the different types of dual thermoresponsive nanoparticles that have been studied, many employ copolymers comprising blocks of poly(*N*‐isopropylacrylamide) (PNIPAAM),^27^, ^30^ poly(ethylene oxide),^31^ and poly(diethylene glycol methyl ether methacrylate),^32^ which possess a lower critical solution temperature (LCST). Such dual thermoresponsive particles typically include two distinct polymer domains, each with a distinct LCST, so that particle size or morphology can be changed at each of the LCSTs.^28^ The transition above or below either LCST can also result in the complete dissociation of the nanoparticle to its constituent assembly units, and thereby act as a spontaneous drug release mechanism.^17^

While many of these dual thermoresponsive polymer nanoparticle systems have been foundational in the field, there are a limited number of polymers with LCSTs in the physiologically‐relevant range, and the few reports that mention manipulation of the LCST require complex chemical modifications that range from the attachment/replacement of specific chemical handles or conjugation with peptides, proteins, or other polymers.^10^, ^33^, ^34^ The synthesis routes for many of these polymers are facilitated by techniques such as reversible addition‐fragmentation chain transfer^29^ and quasi‐living radical polymerization via Ce(IV) ion reduction,^31^ which can require careful selection of specialized monomer and chain‐transfer agents.^35^, ^36^

Nature‐inspired thermoresponsive biomaterials such as recombinantly engineered or chemically synthesized polypeptides serve as good alternatives to synthetic polymers, owing to the defined peptide sequence and chain length, which in turn provide precise control of structural, chemical, and thermodynamic properties.^37^, ^38^ The most common thermoresponsive polypeptides that have been studied in recent years include collagen‐like peptides (CLPs)^39^ and elastin‐like polypeptides (ELPs).^40^ Briefly, CLPs consist of a characteristic (G‐X_AA_‐Y_AA_)_*n*_ amino acid repeat sequence where X_AA_ and Y_AA_ can be any L‐amino acid but are typically the imino acids proline and hydroxyproline (P and O, respectively). Similarly to native collagen protein, individual chains of CLPs with repeat lengths of *n* ≥ 6 form a left‐handed polyproline type II helix, and these strands in turn form a right‐handed triple helix upon alignment of the three individual chains with a one residue stagger between each chain.^41^ The resulting triple helix possesses a characteristic melting temperature (*T*_m_) that is dependent on both the number of G‐P‐O repeats and the amino acid sequence; the effects of such parameters on *T*_m_ have been rigorously investigated by others.^42^, ^43^, ^44^, ^45^ CLPs have been tested for their potential in diagnostic applications,^46^ their ability to form supramolecular structures,^47^, ^48^, ^49^, ^50^ and their properties as components in hydrogels^39^, ^51^ and cell adhesion scaffolds,^52^, ^53^ but CLPs have infrequently been studied as thermoresponsive elements in materials.

Similar to CLPs, ELPs are derived from a mammalian protein, in this case the elastin precursor protein tropoelastin. Most sequences that have been studied possess the characteristic repeat of (V‐P‐G‐X_AA_‐G)_*n*_, where X_AA_ can be any amino acid with the exception of proline.^37^ Like the polymers PNIPAAM and PEG, ELPs possess an LCST‐like transition, also known as the inverse transition temperature (*T*_t_), above which they form a coacervate phase in which they are not soluble and aggregate; upon re‐cooling below the *T*_t_ they resolubilize.^37^ The ELP transition temperature phenomenon is rooted in the dehydration and hydrophobic association of the ELP chains, but like CLP folding/unfolding, is fully reversible.^54^ ELPs have been investigated for their ability to form nanoparticles with promising properties in drug delivery applications.^55^, ^56^ In such applications, most ELP nanoparticles drive the formation of micelles by a variety of mechanisms including electrospraying,^57^ amphiphilic self‐assembly (e.g., using di‐block ELP fusion constructs of differing hydrophobicities),^58^ or conjugation with hydrophobic drug loaded cysteine ligands.^25^ While such assemblies are perhaps more attractive over synthetic polymers due to their innate biocompatibility, they still rely largely on a singular *T*_t_ for assembly and do not fully utilize this thermodynamic parameter for time‐activated release of encapsulated drug molecules.^59^ A recent report has highlighted the recombinant fusion constructs of block ELPs and resilin‐like polypeptides to form a variety of self‐assembled morphologies, though despite the presence of two thermoresponsive blocks, no dual thermoresponsiveness was reported.^60^

We previously reported that the short ELP peptide, (VPGFG)_6_, possesses an experimentally inaccessible *T*_t_ (>80°C in pure aqueous conditions)^61^; conjugating the CLP (GPO)_4_GFOGER(GPO)_4_GG to the ELP enables the ELP to undergo an inverse phase transition and collapse, but only when the CLP domain is in triple helical form. The resulting folded ELP‐CLP trimers, upon coacervation of the ELP domain, were observed to spontaneously self‐assemble into vesicles with a bilayer comprising ELP‐CLP ternary subunits. These vesicles were shown to be stable over a significant temperature range but could dissociate at ~70°C due to the unfolding of the CLP triple helical domain.^61^

Herein, we sought to not only lower this CLP‐mediated hyperthermal dissociation temperature of vesicles to a more clinically approachable value but also determine an underlying set of design rules for the ELP‐CLP vesicles and the role that CLPs have in their self‐assembly. To accomplish this, we synthesized a small library of CLP sequences that have previously been shown to possess *T*_m_ values lower than that of the (GPO)_4_GFOGER(GPO)_4_GG CLP originally studied.^41^, ^47^, ^61^, ^62^, ^63^ The *T*_m_ values of these four CLP sequences, as well as the *T*_m_ values of the corresponding ELP‐CLP conjugates (employing the ELP (VPGFG)_6_ used in our previous study), were determined by circular dichroism (CD) spectroscopy. The *T*_m_ values were then used to predict the self‐assembly (or lack thereof) and thermal responsiveness of the ELP‐CLP conjugates, and the resulting structures of the ELP‐CLP assemblies were characterized via dynamic light scattering (DLS), turbidity measurements, and transmission electron microscopy (TEM). Our studies highlight the dependence of ELP‐CLP conjugate *T*_t_ values on CLP T_m_ values as well as the hydrophobicity of the CLP. The work here also highlights the possibility of optimizing both the ELP and CLP sequences to create a dual thermoresponsive vesicle system that is biocompatible^62^ and possesses physiologically‐relevant thermoresponsive domains for either hyper‐ or hypothermic treatments.

## 2 MATERIALS AND METHODS

### 2.1 Materials

Dimethylformamide (DMF), methylene chloride, HPLC‐grade water, HPLC‐grade acetonitrile, dimethylsulfoxide (DMSO), anhydrous diethylether (ethyl ether), 0.22 μm (13 mm OD) polyvinylidene difluoride (PVDF) syringe filters, sodium chloride, and Whatman grade 1 filter paper were purchased from Thermo Fisher Scientific (Waltham, MA). Phosphotungstic acid, copper (II) sulfate (Cu(II)sulfate), (+)‐sodium l‐ascorbate, aminoguanidine hydrochloride, trifluoroacetic acid (TFA), triisopropylsilane (TIS), 4‐methylmorpholine, diisopropylethylamine (DIPEA), piperidine, Hellmanex® III cuvette cleaning solution, a Hellma® 45 × 12.5 × 4.5 mm Suprasil® quartz cuvette, and Nunc® MicroWell® MiniTrays were purchased from Sigma‐Aldrich, Inc. (St. Louis, MO). Tris‐hydroxypropyltriazolylmethylamine (THPTA) was purchased from Click Chemistry Tools LLC (Scottsdale, AZ). Activating reagent *N*‐[(1H‐benzotriazol‐1‐yl) (dimethylamino)methylene]‐*N*‐methyl‐methan‐aminium hexafluorophosphate *N*‐oxide (HBTU) and common Fmoc protected amino acids such as Fmoc‐l‐glycine‐OH, Fmoc‐l‐proline‐OH, Fmoc‐l‐hydroxyproline(t‐butyl)‐OH, Fmoc‐_L_‐valine‐OH, Fmoc‐_L_‐phenylalanine‐OH, Fmoc‐l‐glutamic acid(t‐butyl)‐OH, and Fmoc‐l‐arginine(Pbf)‐OH were purchased from AAPPTec, LLC (Louisville, KY). Specialty amino acids 4‐azidobutyric acid and Fmoc‐l‐propargylglycine‐OH were purchased from ChemPep Inc. (Wellington, FL). Low loading (LL) Rink Amide ProTide® Resin was purchased from CEM Corporation (Matthews, NC).

### 2.2 Synthesis and characterization of elastin‐like and collagen‐like peptides

The CLP peptides N_3_‐(GPO)_6_GG, N_3_‐(GPO)_7_GG, N_3_‐(GPP)_10_GG, N_3_‐(GPO)_3_GFOGER(GPO)_3_GG (N_3_ denotes the N‐terminal azide, 4‐azidobutyric acid) and the ELP peptide NH_2_‐(VPGFG)_6_G′ (G′ denotes the alkyne‐containing propargyl glycine) were synthesized using the standard Fmoc/t‐butyl solid phase peptide synthesis strategy on a Tribute® automatic peptide synthesizer (Gyros Protein Technologies, Tucson, AZ). Specific details of the peptide synthesis, cleavage, purification, and mass spectrometry protocols that were used can be found in Supporting Information Section 1.1. Total ion chromatograms and mass spectra of the peptides are given in Figures S1–S6.

### 2.3 Synthesis and characterization of ELP‐CLP conjugates

The ELP‐CLP conjugates (VPGFG)_6_‐(GPO)_6_GG (denoted [F6‐GPO6]), (VPGFG)_6_‐(GPO)_7_GG, (denoted [F6‐GPO7]), (VPGFG)_6_‐(GPO)_3_GFOGER(GPO)_3_GG (denoted [F6‐GFOGER]), and (VPGFG)_6_‐(GPP)_10_ (denoted [F6‐GPP10]) were synthesized using an adaptation of the common copper (I)‐catalyzed azide‐alkyne cycloaddition (CuAAC) reaction protocol that has been summarized by Presolski et al.^64^ Specific details of the reaction, purification, and mass spectrometry protocols can be found in Supporting Information Section 1.2. Total ion chromatograms and mass spectra are presented in Figures S7–S11. Conjugates were further characterized using Fourier transform infrared spectroscopy (FTIR); these data are summarized in Figure S12 along with corresponding experimental conditions that are provided in Supporting Information Section 1.3.

### 2.4 CD spectroscopy of peptides and conjugates

In order to characterize triple helix formation, CD spectroscopy was performed on all peptides and conjugates. All CD experiments were performed using a Jasco 810 CD spectropolarimeter (Jasco, Easton, MD) with a 0.2 cm path length cuvette. Detailed experimental procedures of CD measurements and calculations are provided in Supporting Information, Section 1.4. All peptide (CLP and ELP only, not conjugates) experiments were performed at a concentration of 0.35 mM in HPLC grade water (pH 6.5 at 4°C). Conjugate wavelength scans were performed at a concentration of 0.1 mM in HPLC grade water and conjugate melting temperature scans were performed at a concentration of 0.35 mM in HPLC grade water (pH 6.5 at 4°C). The molar concentrations for both the CLPs and the ELP‐CLP conjugates were identical in order to ensure accurate comparisons between the melting temperatures of the CLP in its free and conjugated forms. It should briefly be mentioned that during the melting temperature scan for the F6‐GPO7 conjugate, there was a modest drop in the measured signal (voltage) due to the increased scattering by the vesicles; However, there was still sufficient signal for an accurate melting temperature trend to be fit to the data. This was not of issue for the F6‐GPO6 and F6‐GFOGER conjugates, as these sequences did not self‐assemble under the experimental conditions. Conversely, a melting temperature scan could not be performed for F6‐GPP10 due to significant aggregation and settling. All wavelength scan data (at 4 and 80°C) are provided in Figures S13, S14a, S15, and S16 (CLP, ELP, conjugates excluding F6‐GPP10, and F6‐GPP10 respectively). The ELP melting curve scan is provided in Figure S14b and all CLP melting curves are provided in Figure S17. The melting temperature for all CLPs and ELP‐CLP conjugates was defined as the minima of the first derivative of a Boltzmann function that was fit to the melting curve transition between 4 and 80°C. The fitting of the melting curves using the sigmoidal Boltzmann function was completed using Origin 2017 software (Originlab Corporation, Northampton, MA).

### 2.5 Preparation and characterization of ELP‐CLP vesicles via dynamic light scattering

#### 2.5.1 Vesicle formation

For vesicle formation, conjugates were first dissolved into a 1 mg/ml solution of either pure HPLC‐grade water or 100 mM NaCl aqueous solution. In all cases, the conjugate solution was capped and wrapped in parafilm and placed in an 80°C oven for ~2 hr. A prerinsed and precleaned 10 mm pathlength cuvette, 1 ml syringe, and 0.22 μm PVDF syringe filter were also incubated in the same oven during the final 30 min of conjugate solution incubation. After incubation, 1 mL of conjugate solution (80°C) was filtered at 80°C directly into the cuvette, which was subsequently capped and wrapped in parafilm before being placed in the DLS instrument (Malvern Panalytical Inc., Westborough, MA), with the DLS cuvette well preset to a temperature of 80°C.

Vesicle formation was induced by cooling the sample in a controlled manner in the cuvette well. The cooling steps proceeded in the following sequence: 80, 65, 50, 35, 30 (only for F6‐GFOGER), and 25°C, with a 5‐min equilibration at each temperature followed by three light scattering measurements. A non‐negative least squares analysis of the autocorrelation function was employed by the Malvern software to derive a set of translational diffusion coefficients for each measurement, which were then converted to the number average (n‐ave) diameters by utilizing the Stokes‐Einstein equation as has been previously reported.^65^ For this analysis, a scattering angle of 173° was employed, the refractive index (RI) for the ELP‐CLP was assumed to be that of a protein (RI = 1.45), and the refractive index of water was set to be RI = 1.33. In all cases, automatic attenuation and measurement position were utilized so that the detector would not become saturated with light as vesicles began to form. Each light scattering measurement also incorporated a correlation delay time of 10 s and a sub‐run count of 20 correlations for each scattering measurement. Figure S18a–d shows the representative autocorrelation functions for each temperature for each conjugate. In the cases where vesicle formation occurred, the vesicles were incubated in the cuvette well at 25°C overnight prior to characterization via TEM or determination of the inverse transition temperature.

#### 2.5.2 Vesicle inverse transition temperature determination

In order to determine the diameter of vesicles through the inverse transition temperature process for a given conjugate, the solution of vesicles was slowly cooled in 5°C intervals from 25 to 5°C, with each temperature setting employing a 5‐min equilibration step followed by three light scattering measurements. As before, automatic attenuation and measurement position were employed. The scattering angle and refractive indices of the sample and water employed were the same as those noted in Section 2.5.1. The autocorrelation function was recorded with a 10 s correlation delay time with collection of 20 sub‐run correlation functions for each measurement. Each set of autocorrelation data was analyzed using the nonlinear least squares Malvern algorithm and the Stokes‐Einstein equation as described above.

### 2.6 TEM of ELP‐CLP vesicles

All of the vesicles were prepared for TEM in a similar fashion with either a single staining protocol or a triple staining protocol that was used to enable observation of the vesicle bilayer. Carbon‐coated copper grids were ionized with a PELCO easiGlow® (Ted Pella Inc., Redding, CA) glow discharge unit to make the grids more hydrophilic prior to grid spotting. After overnight incubation of the sample at 25°C, 18 μl of 1 mg/ml vesicles were pipetted into a single well of a Nunc® MicroWell® Mini Tray. A freshly ionized grid was then gently rested on the surface of the vesicle droplet for 1 min, after which the grids were stained with phosphotungstic acid (1 wt% solution) and subsequently washed and blotted; detailed protocols are provided in Supporting Information Section 1.5. All grids were observed with a Carl Zeiss Libra 120 TEM with a high‐tension voltage of 120 V. Vesicle diameters and bilayer lengths were quantified using the ImageJ software (NIH, Bethesda, MD).^66^

Identical grid spotting procedures were employed for the F6‐GPO7 conjugate at higher temperatures. The analysis of F6‐GPO7 vesicle morphologies at the higher temperatures (50, 65, and 80°C) required incubation and equilibration of all materials (Nunc® MicroWell® MiniTray, 1 mg/ml vesicle solution, 20 μl pipette tips, preionized grids, and 1 wt% PTA stain) in a Heratherm Oven (Thermo Fisher Scientific, Waltham, MA) at the corresponding temperature for 20 min prior to grid spotting, which was subsequently performed inside the oven. Analysis of vesicle morphologies at the 4°C temperature required incubation and equilibration of all materials (listed above) within a 4°C cold room for 20 min, with the additional requirement that the vesicle and staining solutions were kept on ice until being distributed to the well plate for spotting.

### 2.7 Turbidity measurements of ELP‐CLP conjugate vesicles and aggregates

#### 2.7.1 Turbidity measurements of F6‐GPO7 vesicles

Additional characterization of the vesicle inverse transition temperature was performed by UV‐Vis mediated detection of turbidity of vesicle solutions as a function of temperature. Directly after vesicle formation, a 1 mg/ml solution of vesicles was inserted into the cuvette well of a Cary 60 UV‐Vis spectrophotometer equipped with a temperature and magnetic stirring controller as well as a nitrogen gas line to constantly purge the cuvette well of condensing water vapor. To determine the inverse transition temperature, the turbidity of the F6‐GPO7 vesicle solution was continuously monitored with a 600 nm laser over the course of cooling from 25 to 0°C with a cooling rate of 1°C/min. The inverse transition temperature was defined as the temperature at which the turbidity was equal to that of 50% maximal turbidity, as in previous studies.^37^

To assess the reversibility of the dissolution and re‐assembly of vesicles, F6‐GPO7 vesicles were repeatedly thermally cycled from 25 to 0°C and back to 25°C, with continuous monitoring at 600 nm in a Cary 60 UV‐Vis spectrophotometer over the course of 125 min while the cuvette temperature controller was cycled (and incubated) between temperatures of 25 or 0°C. The dead‐time for cooling from 25 to 0°C was ~5 min, while the dead‐time for heating was ~1.5 min. In between heating and cooling steps, the 25 or 0°C temperature was held on average for 3 min while the turbidity or lack thereof was monitored.

#### 2.7.2 Turbidity measurements of F6‐GPP10 aggregates

The attempted particle formation procedure for F6‐GPP10 resulted in bulk aggregates. To assess the thermal responsiveness of these aggregates, turbidity measurements were performed with heating or cooling temperature gradients. To assess the hyperthermal mediated re‐dissolution of the F6‐GPP10 aggregates, a 1 mg/ml suspension of aggregates was heated in cuvette with a stir bar in a Cary 60 UV‐Vis instrument with a 400 rpm stirring rate and a 2.5°C/min heating rate while being simultaneously monitored for turbidity with a 600 nm laser. For determining the inverse transition temperature, the F6‐GPP10 aggregates were again suspended with stirring at 400 rpm and cooled from 25 to 0°C using a 1°C/min cooling rate. The bulk hyperthermal dissolution temperature and the hypothermal inverse transition temperatures for this conjugate were defined as the temperature where a 50% reduction in the maximal turbidity occurred.

## 3 RESULTS AND DISCUSSION

### 3.1 Synthesis and characterization of peptides

In this report, we seek to provide insight regarding the role of the folding and thermal stability of the CLP domain on the inverse transition temperature of the ELP domain and the self‐assembly of ELP‐CLP conjugates relative to the conjugate (VPGFG)_6_‐(GPO)_4_GFOGER(GPO)_4_GG of our previous studies.^61^, ^62^ This conjugate exhibited, as measured via CD spectroscopy, a melting temperature of 57°C, while the unconjugated CLP domain (N_3_‐(GPO)_4_GFOGER(GPO)_4_GG) exhibited a melting temperature of 50°C. Given that the resulting vesicles formed from this conjugate underwent complete thermal dissociation only at relatively high temperatures (70°C),^61^ we employed in the current study CLPs that have previously been shown to possess *T*_m_ values lower than that of the N_3_‐(GPO)_4_GFOGER(GPO)_4_GG sequence.^41^, ^47^, ^63^, ^67^ It is well established that CLP sequences that possess few hydroxyproline residues (O) in the Y_AA_ position, as well as sequences that have relatively short repeat lengths (<8), have lower *T*_m_ values than CLP sequences that are high in O content and/or are relatively long.^41^, ^42^, ^44^, ^67^ For these reasons, four CLPs with an N‐terminal azide (N_3_) (N_3_‐(GPO)_6_GG, N_3_‐(GPO)_7_GG, N_3_‐(GPO)_3_GFOGER(GPO)_3_GG, and N_3_‐(GPP)_10_GG) were chosen for the present study. The (GPO)_3_GFOGER(GPO)_3_GG sequence, owing to its shorter length, exhibits a lower *T*_m_ relative to the sequence in our previous studies, and the (GPO)_*n*_ sequences were chosen as they would be good comparators to this sequence without the inclusion of GFOGER residues.^62^ The (GPP)_10_ sequence was chosen to study the role of chain length and hydrophilicity on the self‐assembly of the vesicles with a CLP domain that exhibits a relatively low *T*_m_.^41^, ^67^ For suitable comparison to our previous work, the same ELP ((VPGFG)_6_G′) with a C‐terminal propargyl glycine (labeled G′) was employed as the ELP component for all of the ELP‐CLP conjugates that were made. All peptides were prepared via solid‐phase peptide synthesis procedures and purified via reverse phase high performance liquid chromatography (RP‐HPLC) (Supporting Information Section 1.1). Ultra‐performance liquid chromatography mass spectroscopy (UPLC‐MS) and corresponding electrospray ionization mass spectra of each purified peptide provided confirmation that the synthesized peptides were pure products (Figures S1–S6).

CD spectroscopy wavelength and temperature scans were performed on all pure ELP and CLP products. The ELP wavelength scans and temperature scan are provided in Figure S14. It should briefly be noted that there is no significant positive contribution at 225 nm from the ELP at 4°C and only a minor negative ellipticity at this wavelength at 80°C (Figure S14a). Additionally, the ellipticity change of the ELP at 225 nm through the course of heating from 4 to 80°C is linear and is not representative of a secondary structure transition (Figure S14b).^68^ This result was anticipated given that this short ELP has previously been shown to possess a *T*_t_ greater than 80°C.^61^

The CLPs were shown to form triple helices that in turn could subsequently be unfolded upon heating (Figures S13 and S17). The experimentally determined melting temperatures for each CLP are delineated in Table 1. As expected, N_3_‐(GPO)_7_GG and N_3_‐(GPP)_10_GG exhibited the highest *T*_m_ values and (GPO)_6_GG the lowest *T*_m_ value, with N_3_‐(GPO)_3_GFOGER(GPO)_3_GG exhibiting an intermediate value owing to its longer repeat length but also to the destabilizing nonimino acid residues in the center of the sequence.^69^ Our results are consistent with the expected behavior of these sequences, although direct comparisons of *T*_m_ values to those reported in the literature are complicated by the fact that triple helical melting temperatures are dependent on both concentration and heating rate.^47^, ^63^, ^67^ Nevertheless, the *T*_m_ values in Table 1 suggest that self‐assembly of these ELP‐CLP conjugates should be possible over a temperature range (27–45°C) that is physiologically and therapeutically relevant.^70^ Furthermore, two conjugates exhibiting nearly the same *T*_m_ (e.g., ((GPO)_7_ and (GPP)_10_)) facilitate comparisons between the self‐assembly of conjugates with different CLP chain lengths and/or hydrophobicities.

**Table 1:** Melting temperatures of purified N‐terminally azide‐functionalized CLPs as determined by CD spectroscopy

CLP sequence	*T*_m_ (°C)
N_3_‐(GPO)_6_GG	27
N_3_‐(GPO)_3_GFOGER(GPO)_3_GG	32
N_3_‐(GPO)_7_GG	44
N_3_‐(GPP)_10_GG	45

*Note*: All peptides were measured in a 0.2 cm cuvette, in HPLC grade water (pH 6.5) at a concentration of 0.35 mM, with a scanning rate of 10°C/hr.

### 3.2 Synthesis and characterization of ELP‐CLP conjugates

ELP‐CLP conjugates F6‐GPO6, F6‐GPO7, F6‐GFOGER, and F6‐GPP10 were synthesized by reacting the purified ELP and CLP components using the standard copper‐catalyzed CuAAC reaction. The ELP‐CLP conjugates were subsequently purified via RP‐HPLC and analyzed via UPLC‐MS methods that generated elution profiles and corresponding mass spectra that confirmed the purity of each conjugate (Figures S7–S11). Confirmation of successful purification of the ELP‐CLP conjugates was further provided from FTIR absorbance spectra which verified the disappearance of the CLP azide chemical functionality of the ELP‐CLP conjugates (Figure S12).

As we have previously reported, the formation of the CLP triple helix is a critical step that enables the self‐assembly of the ELP‐CLP vesicles.^61^ CD spectroscopy wavelength scans were therefore conducted on the purified conjugates to ensure triple helix formation (Figures S15 and S16). It should briefly be noted here that the spectra of the ELP‐CLP conjugates in Figures S15 and S16 correspond distinctly to triple helices. Though the existence of β‐turn secondary structures for ELPs in the collapsed state has been reported previously, we show no conclusive evidence for their presence in the CD spectra of the conjugates reported here, most likely due to the considerably weaker absorption of β‐turns relative to the absorption of triple helices, β‐sheets, and α‐helices.^71^ No transitions were observed in the ELP domains alone (Figure S14), as the ELP does not undergo a thermally‐induced coacervation as previously reported.^61^

**Figure 1:**
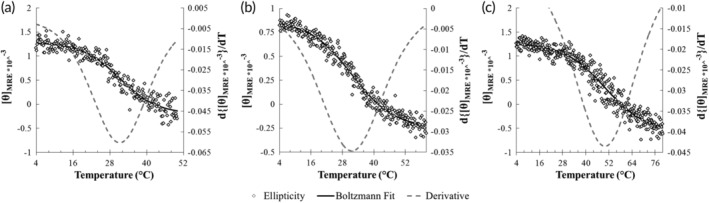
Circular dichroism melting temperature scans at 225 nm of ELP‐CLP conjugates: (a) F6‐GPO6, (b) F6‐GFOGER, and (c) F6‐GPO7. Mean residue ellipticity ([θMRE]) is shown for each conjugate in white (black outlined) diamonds, the Boltzmann sigmoidal fits of the ellipticity data are given by black solid lines, and the first derivatives of the Boltzmann fits are illustrated by gray dashed lines. The melting temperature for each conjugate is defined to be the minima of the first derivative of the Boltzmann fit, and corresponds to 31, 32, and 50 for (a), (b), and (c) respectively

The melting temperature of each of the ELP‐CLP conjugates (with the exception of F6‐GPP10) was determined by temperature scans that are shown in Figure 1. The *T*_m_ of the conjugates showed the same stability trends as the corresponding CLP domains alone, with values of 31, 32, and 50°C for F6‐GPO6, F6‐GFOGER, and F6‐GPO7, respectively (Table 2). The melting temperature of F6‐GPP10 could not be determined quantitatively given its precipitation at lower temperatures, but was estimated to be between 50 and 62.5°C given the N_3_‐(GPP)_10_GG melting temperature (Table 1) and turbidity data (see Section 3.5 below).

**Table 2:** Melting temperatures of purified ELP‐CLP conjugates as determined by CD spectroscopy

Designation	Conjugate sequence	*T*_m_ (°C)
F6‐GPO6	(VPGFG)_6_‐(GPO)_6_GG	31
F6‐GFOGER	(VPGFG)_6_‐(GPO)_3_GFOGER(GPO)_3_GG	32
F6‐GPO7	(VPGFG)_6_‐(GPO)_7_GG	50
F6‐GPP10	(VPGFG)_6_‐(GPP)_10_GG	‐

*Note*: All conjugates were measured in pH 6.5 HPLC grade water at a concentration of 0.35 mM with a scanning rate of 10°C/hr in a 0.2 cm cuvette. Note that a *T*_m_ for F6‐GPP10 could not be obtained due to its aggregation.

The melting temperatures of both F6‐GPO6 and F6‐GPO7 increased by approximately 4°C and 6°C, respectively, as compared with the values of the corresponding CLPs alone (Figure 1a,c and Tables 1 and 2), consistent with our previous report.^61^ Interestingly, this increase was not observed for the F6‐GFOGER conjugate, which exhibited a similar CLP melting temperature as the CLP domain alone (Figure 1b and Tables 1 and 2). In our original report, the increase in CLP *T*_m_ was ascribed to the ELP chains acting as a hydrophobic anchoring point that partially limited the unfolding of the triple helical domain.^61^ The lack of this observation for the F6‐GFOGER conjugate may suggest that the number of (GPO) repeat units proximal to the ELP is important given the fact that GPO repeats are the strongest triple helical formers out of all other tripeptides.^69^, ^72^ Regardless, the CD data suggest that for F6‐GPO7 and F6‐GPP10, it should be possible to initiate ELP‐CLP assembly at temperatures below or proximal to 50°C, while for the F6‐GFOGER and F6‐GPO6, assembly should occur near 32 and 31°C, respectively.

### 3.3 Formation and characterization of ELP‐CLP vesicles

**Figure 2:**
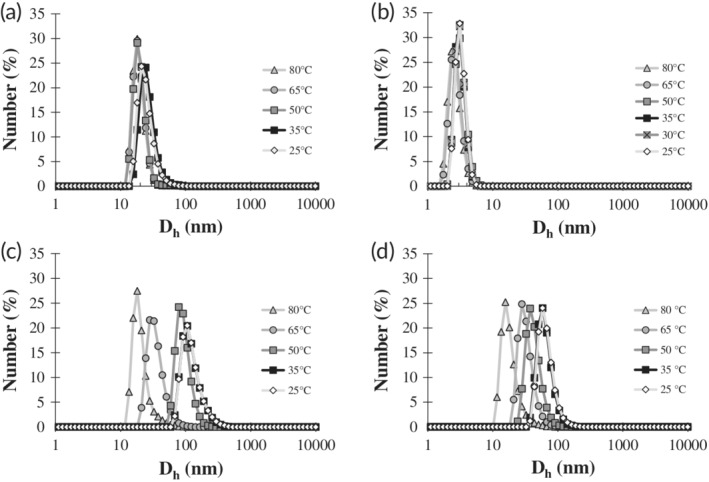
Number averaged hydrodynamic diameter distribution profiles of ELP‐CLP conjugates as a function of temperature that were obtained by DLS. Panels correspond to the following ELP‐CLP conjugates: (a) F6‐GPO6, (b) F6‐GFOGER, (c) F6‐GPO7, and (d) F6‐GPP10. Each conjugate (1 mg/ml in deionized water) was heated to 80°C and subsequently cooled and measured at 80°C (light gray line and triangle), 65°C (gray line and circle), 50°C (dark gray line and square), 35°C (black line and square), and 25°C (light gray line and white diamond). The F6‐GFOGER had an additional cooling stage at 30°C (gray line with × marked gray square). Only the F6‐GPO7 and F6‐GPP10 were observed to have any increases in size during cooling, indicating that only these conjugates could self‐assemble. The number average hydrodynamic diameters of F6‐GPO7 and F6‐GPP10 vesicles at 25°C were 104 ± 5 nm and 60 ± 10 nm, respectively. The standard error reported is based upon analysis of multiple batches of vesicles (*n* = 3 for F6‐GPO7 and *n* = 2 for F6‐GPP10)

In order to form vesicles, purified ELP‐CLP conjugates were dissolved in pure deionized water, heated to 80°C, and equilibrated for 2 hr so that the CLP domains of each conjugate would be sufficiently unfolded and the conjugate molecules dissolved in solution (Figures S15b and S16). Vesicle formation was conducted directly in a cuvette via step‐wise controlled cooling steps (80, 65, 50, 35, and 25°C) in the DLS instrument so that vesicle formation could be observed *in situ*. It is worth briefly noting that this vesicle formation process is significantly more facile than other vesicle formation techniques that require specific pH aqueous environments,^6^ organic to aqueous solvent exchanges,^8^ or specific instrumentation (e.g., spray drying).^73^ The hydrodynamic diameter distributions derived from raw autocorrelation decay functions for each conjugate sequence were recorded at each temperature throughout the cooling steps (Figures 2 and S18). The average hydrodynamic diameters for the vesicles formed from F6‐GPO7 and F6‐GPP10 at each temperature are provided in Table S1.

The data in Figure 2 demonstrate that the hydrodynamic diameter (*D*_h_) distributions at 80°C for all the conjugates were approximately within the expected range of the estimated length of a single ELP‐CLP subunit. This suggests that all of the conjugates adopted their monomeric form as nontriple helical sub‐units at 80°C, which is consistent with our previous reports.^61^, ^62^ In the case of the F6‐GPO7 and F6‐GPP10 conjugates, the correlation delay times increased as cooling proceeded, indicating distributions with higher *D*_h_ values, and suggesting the formation of vesicles (Figure 2c,d). The temperature at which the assembly began to occur correlated well with the measured *T*_m_ values determined by CD (Figure 1 and Table 2), corroborating that the triple helical folding of the CLP domain in the ELP‐CLP conjugates is necessary for the self‐assembly of these conjugates into vesicles.

The lack of any significant increases in the hydrodynamic diameter of the F6‐GPO6 and F6‐GFOGER conjugates as temperature decreased showed that these conjugates did not form vesicles (Figure 2a,b). We briefly note that the size distributions of the F6‐GPO6 and F6‐GFOGER at all temperatures appear to be modestly different from each other, though these subtle differences are likely not significant given the limitations of the characterization method. These F6‐GPO6 and F6‐GFOGER conjugates possess significantly lower melting temperatures (by ~20°C) than F6‐GPO7 and F6‐GPP10 but were still shown to be capable of forming triple helices (Table 2, Figures 1, and S15). As such, it was expected that some degree of assembly would occur around the melting temperatures for these conjugates, similar to what was observed at 50°C for F6‐GPO7 and F6‐GPP10 (Figure 2c,d). It was postulated that the self‐assembly of the previously reported (VPGFG)_6_‐(GPO)_4_GFOGER(GPO)_4_GG conjugate occurred due to the reduction in the inverse transition temperature and collapse of the ELP domain that was facilitated by the increase in the local concentration of ELPs upon formation of the CLP triple helix.^61^ This rationale was made based on ample ELP literature, which has shown that the *T*_t_ is a function of ELP concentration (e.g., a reduction in *T*_t_ is a result of increased ELP concentration and vice versa).^54^, ^74^ However, based on our current observations of the F6‐GPO6 and F6‐GFOGER conjugates, which clearly fold into triple helices (Figures 1, S15, and S16) but yet do not form assembled structures at temperatures near or below their *T*_m,_ we suggest that changes in the local concentration alone are not sufficient to drive assembly. Indeed, when comparing the % of triple helical folding for F6‐GPO7, F6‐GPO6, and F6‐GFOGER (Table S2), although each conjugate was ~50% folded at its *T*_m_, only the F6‐GPO7 conjugate was observed to begin self‐assembly (Figure 2c).

The data in this work indicate that the differing triple helical “strengths” of CLP folding (i.e., the different *T*_m_ values of the CLPs) affect the magnitude of the shift in the ELP *T*_t_ values during the transition between the monomeric and the CLP‐conjugated and folded (trimeric) forms. This likely results from differences in the strength of the interactions of the CLP domains that consequently affect local ELP chain interactions and hydration state, and so modify the ELP transition and collapse.^67^, ^74^ Since the F6‐GPO6 and F6‐GFOGER conjugates do not form vesicles but do form triple helices, their respective *T*_t_ values must be greater than their *T*_m_ values, and thus the ELP domains do not undergo coacervation at a temperature at which the CLP domain forms a triple helix. The ability of the ELP domain to undergo coacervation in the triple‐helical ELP‐CLP form was confirmed by studying the vesicle assembly of F6‐GFOGER in an ionic solution (e.g., 100 mM NaCl), as the addition of salt is a known method of lowering the *T*_t_,^74^, ^75^ and the effect is magnified when the ELP contains a charged residue such as glutamic acid or arginine, thus motivating the study of this condition for the F6‐GFOGER conjugate.^76^ We find via DLS and TEM that the F6‐GFOGER conjugate assembles into vesicles when cooled to approximately its *T*_m_ (Table 2) in the presence of 100 mM NaCl (Figures S19 and S20), but not in a solution of pure water (Figure 2b). These results suggest that the coacervation of the ELP domain in F6‐GFOGER is indeed possible, but that the stability of the CLP domain is insufficient (and/or the triple‐helical conjugate is too hydrophilic) for this coacervation to occur in deionized water. Details of this experiment are provided in Supporting Information Section 1.6.

In contrast to the F6‐GPO6 and F6‐GFOGER conjugates, the F6‐GPO7 and F6‐GPP10 conjugates do self‐assemble, indicating *T*_t_ values for these conjugates are less than their respective *T*_m_ values and are likely experimentally accessible between 0°C (freezing point of water) and ~50°C (the *T*_m_ and estimated *T*_m_ of F6‐GPO7 and F6‐GPP10, respectively). Furthermore, the fact that not all ELP‐CLP conjugates form vesicles at temperatures below their *T*_m_ implies that there may be some critical *T*_m_ at which the *T*_t_ of a given ELP domain shifts sufficiently such that self‐assembly can occur. In the context of this work, we speculate that this critical *T*_m_ is between 32 and 50°C.

### 3.4 Dual thermoresponsiveness and morphological characterization of F6‐GPO7 vesicles

The two conjugates (F6‐GPO7 and F6‐GPP10) which formed vesicles in water were monitored for an extended period of time at 25°C via DLS to ensure that the formed aggregates were colloidally stable over the timescales of the reported experiments. Continuous DLS analysis of the F6‐GPO7 vesicle solution was carried out over a 34‐hr period at 25°C, and throughout that time the hydrodynamic diameter, as well as the attenuation‐corrected photon count rate, remained relatively constant and the vesicles in solution appeared homogenous, well dispersed, and stable (Figure S21).

**Figure 3:**
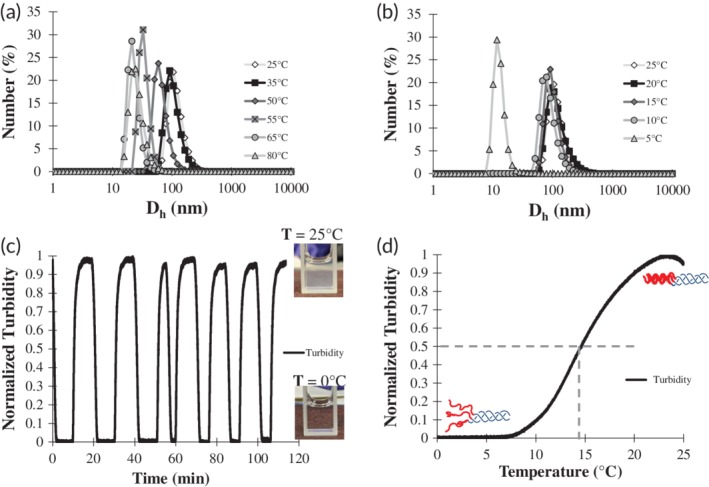
Dual thermoresponsivness of F6‐GPO7 vesicles. (a) Number average *D*_h_ distribution profiles of F6‐GPO7 as function of heating to determine the reversibility of vesicle formation/dissolution. The F6‐GPO7 vesicles were heated from 25°C (light gray line and white diamond) to 35°C (black line and square), 50°C (dark gray line and diamond), 55°C (gray line and x marked gray square), 65°C (gray line and circle), and lastly 80°C (light gray line and triangle). (b) Number averaged *D*_h_ distribution profiles of F6‐GPO7 as a function of cooling to determine the existence of an *T*_t_ for F6‐GPO7. F6‐GPO7 vesicles were cooled from 25°C (light gray line and white diamond) to 20°C (black line and square), 15°C (dark gray line and diamond), 10°C (gray line and circle), and lastly 5°C (light gray line and triangle). (c) Normalized turbidity cycling profiles of the F6‐GPO7 vesicles as the temperature was cooled from 25 to 0°C and then subsequently reheated to 25°C after a short holding period of ~3 min at each end‐point temperature. Seven cycles in total were performed confirming the robust reversibility of the apparent F6‐GPO7 *T*_t_ phenomena. (d) Normalized turbidity of F6‐GPO7 vesicles that was measured as a function of a 1°C/min cooling rate in order to determine the *T*_t_ which was defined as the temperature in which 50% of maximal turbidity is reached (marked by dashed gray lines)

To ensure that there was no significant hysteresis in assembly and disassembly, the F6‐GPO7 vesicles were heated over the same temperature range at which they were formed. Figure 3a shows the corresponding *D*_h_ distributions throughout the heating process. Similar to the cooling distributions in Figure 2c, the heating distributions showed intact vesicles present at 25 and 35°C, but that there is a significant decrease in *D*_h_ at the CLP melting temperature of 50°C. With a further increase in temperature to 55°C, substantial reductions of the *D*_h_ took place; the *D*_h_ values did not change further at 65°C and 80°C, indicating that the vesicles had largely dissociated at temperatures just above the *T*_m_. The similarity in the vesicle size distributions during cooling and heating suggested that vesicle formation and dissociation were reversible, as might be expected based on the reversibility of the inverse temperature transition of ELPs.^54^, ^74^ This observation suggests that transitions in the structure and solubility of the CLP and ELP are both occurring in the dissociation event, with the CLP unfolding first followed by the substantive increase of the ELP *T*_t_ from its trimeric value to its monomeric value, ultimately resulting in vesicle dissociation and solubilization.

The possibility that the F6‐GPO7 conjugate also possesses an experimentally accessible *T*_t_ was assessed via DLS experiments, by monitoring changes in vesicle size as a function of incremental cooling. As illustrated in Figure 3b, the similarity of the *D*_h_ distributions of the F6‐GPO7 conjugate upon cooling from 25 to 10°C indicated no significant change in vesicle size, although a significant drop in the average *D*_h_ was observed upon cooling from 10 to 5°C (Figures 3b and S22a). The shift in *D*_h_ from approximately 100 nm to 20 ± 10 nm (see also Figure S22b) was similar to that of the observed dissociation of vesicles at temperatures greater than 50°C (Figure 3a and Table S1), suggesting an ELP‐mediated dissociation event was taking place since: (a) the CLP triple helix is still folded at these temperatures (Figure 1c) and (b) the change was rapid (within a 5°C window), which is consistent with the rapid nature of ELP coacervation and resolubilization.^37^ The reversibility of this transition was assessed via UV‐Vis analysis of the turbidity of the vesicle dispersion over a minimum of 7 cycles; the results shown in Figure 3c indicated that upon cooling to 0°C, the normalized turbidity of the vesicles diminishes to that of water (bottom image in Figure 3c); meanwhile, when the solution was reheated to 25°C, the solubilized triple helical F6‐GPO7 conjugates readily reassembled to their original turbidity (top image in Figure 3c). These data highlight the reversibility and speed of the vesicle dissolution/formation event, consistent, as noted above, with the expected phase transition of the ELP domain.^37^, ^74^

The apparent *T*_t_ of the F6‐GPO7 conjugate was also assessed via these turbidity measurements,^37^, ^77^ by monitoring turbidity during cooling (at 1°C/min) of the solution from 25 to 0°C. The resulting turbidity data are plotted in Figure 3d along with corresponding illustrations of the proposed structures of the conjugates above and below the *T*_t_. The inverse transition temperature (defined as the temperature of 50% of maximal turbidity as is common convention for ELP coacervation) for the F6‐GPO7 conjugate was 14.6°C (Figure 3d).^37^, ^77^ It should briefly be noted that while the DLS *D*_h_ measurement did suggest the presence of vesicles at 10°C, the size distribution at 10°C was slightly lower than all other temperatures (Figure 3b). Given the sensitivity of DLS measurements and the shift in the size distribution, it is possible that vesicles are present at 10°C, but at low concentrations.^78^ Regardless, the 14.6°C *T*_t_ is sufficiently below the *T*_m_ values (ca. 30°C) of the F6‐GPO6 and F6‐GFOGER that assembly would be expected to occur, particularly if the *T*_t_ were strictly a function of the localization of multiple chains of the F6 at the terminus of a CLP triple helix. Thus the *T*_t_ data here further highlight the role of the stability and hydrophilicity of the of the CLP domain in controlling the *T*_t_ of the ELP. In addition, although the *T*_t_ of the F6‐GPO7 is lower than what can be safely applied for cooling deep tissue,^79^ these data provide the first empirical evidence that ELP‐CLP vesicles can exhibit dual thermoresponsiveness that could be employed for drug delivery.

**Figure 4:**
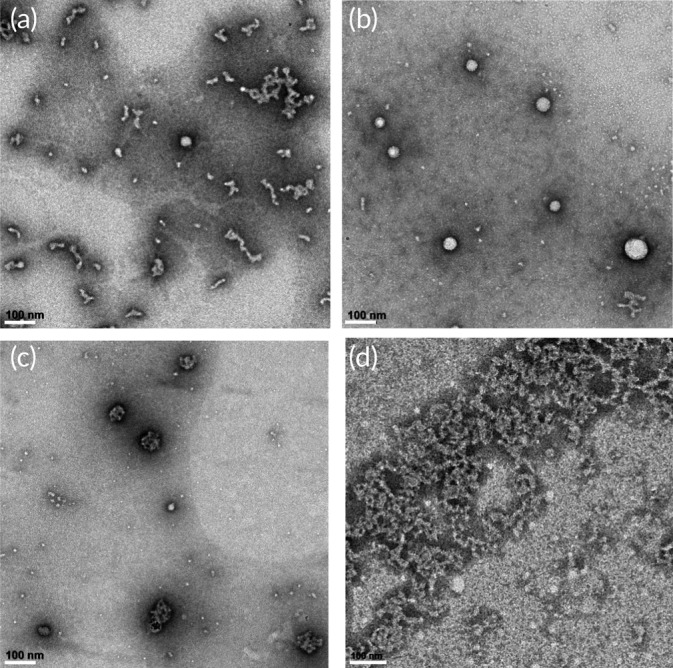
Transmission electron microscopy images of dually thermoresponsive F6‐GPO7 vesicles that were prepared at the temperatures: (a) 4°C, (b) 25°C, (c) 50°C, and (d) 80°C. All vesicles were stained with 1 wt% PTA following the methods described in Section 2.6. All scale bars are equal to 100 nm. The images highlight the dual thermoresponsive nature of the F6‐GPO7 vesicles. The diameters of the vesicles or particle like structures of panels (b) and (c), respectively were (b) 41 ± 15 nm (*n* = 97) and (c) 58 ± 13 nm (*n* = 74)

The morphological characteristics of the F6‐GPO7 vesicles were probed by TEM of samples prepared at various temperatures. TEM grids were spotted with vesicle solutions that were prepared at 4, 25, 50, and 80°C, which correspond to trimeric peptide conjugates resulting from dissociated vesicles (*T* < *T*_t_), stable vesicles (*T*_t_ < *T* < *T*_m_), partially melted/dissociated vesicles (*T* ≈ *T*_m_), and monomeric peptide conjugates resulting from dissociated vesicles (*T* > *T*_m_), respectively. Representative TEM images of the vesicles at each of these temperatures are shown in Figure 4a–d. Replicate data of the TEM analyses for all four incubation temperatures are given in Figure S23a–d. Comparing Figure 4a,b, a stark contrast is observed between the dissociated undefined structures at 4°C (Figure 4a) and the well‐defined spherical morphologies of the vesicles at 25°C (Figure 4b, diameter of 41 ± 15 nm), consistent with the DLS and turbidity data in Figure 3b–d. Similar to the TEM experiments of our first report,^61^ the F6‐GPO7 vesicles incubated at 50°C (Figure 4c) appear enlarged (diameter 58 ± 13 nm) relative to the vesicles incubated at 25°C, presumably due to the unfolding of the CLP domains. This change of size, and an apparent increase of PTA staining of the vesicles,^80^ substantiate the onset of vesicle dissociation suggested in Figure 3a, and also are consistent with the *T*_m_ of the F6‐GPO7 indicated via CD (Figure 1c and Table 2). At 80°C, the data in Figure 4d suggest a lack of vesicles likely due to complete unfolding of the triple helix and subsequent vesicle dissociation. The apparent differences in morphology between the solubilized conjugates in Figure 4a and Figure 4d likely results from the intact CLP triple helices that are present at 4°C relative to the completely unfolded, monomeric ELP‐CLP conjugates that are present at 80°C. Further TEM analysis of F6‐GPO7 vesicles incubated at 25°C was conducted using a multi‐step PTA staining procedure (Supporting Information Section 1.5), which resulted in a more facile observation of the bilayer of the vesicles (similar to vesicular bilayer morphologies observed via TEM by others^81^, ^82^, ^83^) (Figure S24); measured bilayer thicknesses were indicated to be 11.5 ± 2 nm, in reasonable agreement with the predicted thickness of an F6‐GPO7 bilayer of 14.1 ± 0.5 nm (see Supporting Information Section 1.7).^41^, ^43^, ^74^, ^84^, ^85^ The observed differences in the vesicle diameters determined at 25°C via TEM (45 ± 14 nm), versus DLS (104 ± 5 nm), are reasonably consistent with differences observed in previous reports,^86^, ^87^ and can occur owing to differences in population sample sizes^88^ and particle hydration.^89^

In contrast to the F6‐GPO7 vesicles that showed colloidal stability over a 24‐hr period (Figure S21), DLS of the F6‐GPP10 nanoparticles indicated instability of the particle solution, showing distinct features of micron‐scale aggregation after 24‐hr incubation at 25°C, and ultimately precipitation (Figure S25).

### 3.5 Turbidity analysis of F6‐GPP10 aggregates

**Figure 5:**
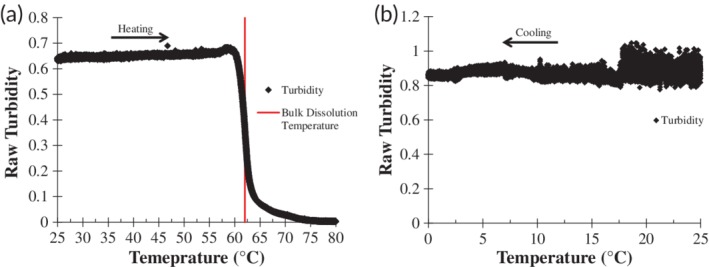
Characterization of F6‐GPP10 aggregates. (a) Turbidity measurements of F6‐GPP10 aggregates (preformed 24 hr prior to measurement (Figure S25)) during heating and stirring (2.5°C/min and 400 rpm, marked by black diamond data points). A red line marks the point in which dissolution of the aggregates reaches 50% of maximal turbidity and the arrow highlights the directionality in which data was recorded (heating). (b) Continuous monitoring with 400 rpm stirring of F6‐GPP10 aggregate turbidity as a function of cooling from 25 to 0°C with 1°C/min cooling rate. The lack of a drop in turbidity indicates that the F6‐GPP10 aggregates do not possess a *T*_t_

Despite the fact that the F6‐GPP10 conjugate precipitated with extended incubation, the temperature‐responsive properties of the F6‐GPP10 aggregates were assessed through turbidity measurements as a function of temperature. Starting with F6‐GPP10 conjugates in the aggregated state (Figure S25), the F6‐GPP10 aggregate solution was stirred (to suspend the aggregates) and heated from 25 to 80°C, crossing the *T*_m_ threshold of the (GPP)_10_ CLP (Table 1 and Figure S17). The turbidity of the suspended aggregates remained relatively constant throughout the heating (Figure 5a), until the temperature reached ~60°C, at which point the turbidity began to decrease rapidly until the solution was clear at temperatures greater than ~75°C due to dissolution of the aggregates. Notably, the onset of the dissociation (at ~60°C) of the F6‐GPP10 conjugate occurred at temperatures that were significantly higher than the *T*_m_ of the N_3_‐(GPP)_10_GG CLP (Table 1 and Figure S17), in contrast to the relatively small difference between the conjugate and CLP *T*_m_ values observed for the other ELP‐CLP conjugates in this study (Tables 1 and 2). This likely occurred both because the *T*_m_ of the F6‐GPP10 conjugate was higher than the *T*_m_ of the N_3_‐GPP10 by itself (following the trend of the other conjugates), and also because of the more rapid heating rate used in this turbidity experiment relative to the slow heating rates used in the CD experiment. Although the turbidity data do not provide a precise *T*_m_ for the F6‐GPP10 conjugate aggregates, they do confirm that folding of the CLP is required for aggregation/assembly to occur.

At the end of the hyperthermal dissolution experiment, the F6‐GPP10 was then cooled and monitored for aggregate formation via turbidity measurements. Detailed analyses of the re‐aggregation of the F6‐GPP10 conjugate and the kinetics of aggregation as well as the morphological characterization of these aggregates is discussed in Supporting Information Section 1.8 and presented in Figure S26. Following the kinetics study and reprecipitation, the F6‐GPP10 aggregates were again suspended with stirring and continuously monitored for turbidity while being cooled from 25 to 0°C to determine if there was a *T*_t_‐mediated dissolution for the F6‐GPP10 conjugate. The data for this experiment are presented in Figure 5b, which shows a continuous unperturbed turbidity signal across the entire temperature window, indicating that the *T*_t_ of the F6‐GPP10 conjugate was less than 0°C.

The highly different assembly/aggregation behavior, despite nearly identical estimated *T*_m_ values, between the F6‐GPO7 and F6‐GPP10 conjugates, suggests that contributions from the final hydration states of the trimerized CLPs can also play an important role in controlling the coacervation behavior of the ELP domain, consistent with the fact that (GPO)‐containing CLPs are moderately more hydrated relative to (GPP)‐containing CLPs.^90^ It previously has been reported that an ELP conjugated with a more hydrophilic moiety (such as *N*‐methyl‐1,6‐OH 1,4,5,6‐tetrahydronicontinamide) will exhibit a higher *T*_t_ than the same ELP conjugated to a less hydrophilic moiety (such as *N*‐methyl‐1,6‐OH 1,4,5,6‐tetrahydronicontinamide).^74^ Thus, perhaps with other important parameters of the ELP‐CLP conjugates being similar (e.g., similar *T*_m_ values), the difference in the hydration of the conjugates may have significant impacts on the *T*_t_. Studies of ELP‐CLP conjugates with CLPs of varying hydrophobicity are necessary to further illuminate the molecular features of this aggregation phenomenon. Nevertheless, taken altogether, our data illustrate the rich range of association/assembly/aggregation behavior that can be accessed in ELP‐CLP conjugates via manipulation of the CLP domain.

## 4 CONCLUSIONS

In this work, we investigated the propensity of four different ELP‐CLP conjugates to self‐assemble into vesicles as a function of the sequence of the CLP domain. The CLP triple‐helix melting temperature (*T*_m_) was found to be a critical parameter that can be used to tune the ELP domain's inverse transition temperature (*T*_t_) from the single chain conjugate state to the trimeric state. The role of *T*_m_ in influencing *T*_t_ and controlling assembly (with the postulated existence of a critical *T*_m_ between roughly 30 and 50°C) was indicated by the lack of vesicle assembly for the F6‐GPO6 and F6‐GFOGER conjugates near their *T*_m_ values, which is in contrast to the formation of well‐defined vesicles by the F6‐GPO7 conjugate near its *T*_m_. Furthermore, of the conjugates studied, only the F6‐GPO7 conjugate exhibits a thermally accessible inverse transition temperature (*T*_t_ = 14.6°C) despite similarities in composition with other conjugates. The rapid and reversible low‐temperature assembly/disassembly of the F6‐GPO7 vesicles is consistent with the behavior of a true inverse transition temperature as reported for ELPs alone.

Our studies also suggest the important role of the hydration of the CLP domain as a parameter controlling ELP‐mediated aggregation/assembly for ELP‐CLP conjugates with a suitably high *T*_m_ such that assembly can occur. Although both F6‐GPO7 and F6‐GPP10 can form assembled/aggregated structures at temperatures below their nearly identical *T*_m_ values, the fact that F6‐GPP10 aggregates could not be solubilized at lower temperatures suggests the importance of the increased hydrophilicity of the GPO‐containing sequences in mediating solubilization of the ELP domains by means of the *T*_t_ phenomena.

The small differences in *T*_m_ but large differences in self‐assembly highlight the importance of the choice of ELP‐CLP pair in the design of dual temperature‐responsive delivery vehicles. The dual‐temperature responsiveness of the F6‐GPO7 conjugate further illustrates the feasibility of designing ELP‐CLP vesicles that can possess both hyperthermic and hypothermic drug release capabilities, with potential future applications in targeted drug release in the treatment of ECM‐related diseases.

## Supporting information


**Appendix** S1: Supporting Information

## References

[1] Kim Y‐J , Matsunaga YT . Thermo‐responsive polymers and their application as smart biomaterials. J Mater Chem B. 2017;5(23):4307‐4321. 10.1039/c7tb00157f 32263961

[2] Stucki M , Stockli M , Stark WJ . Thermoresponsive microspheres as smart pore plugs: self‐venting clothing membranes for smart outdoor textiles. Macromolecular Materials and Engineering. 2018;303(4):1700562.

[3] Kirschner CM , Brennan AB . Bio‐inspired antifouling strategies. Annu Rev Mat Res. 2012;42:211‐229.

[4] Schmidt S , Zeiser M , Hellweg T , Duschl C , Fery A , Mohwald H . Adhesion and mechanical properties of PNIPAM microgel films and their potential use as switchable cell culture substrates. Adv Funct Mater. 2010;20(19):3235‐3243.

[5] Boyer C , Whittaker MR , Luzon M , Davis TP . Design and synthesis of dual Thermoresponsive and antifouling hybrid polymer/gold nanoparticles. Macromolecules. 2009;42(18):6917‐6926.

[6] Zhang LY , Guo R , Yang M , Jiang XQ , Liu BR . Thermo and pH dual‐responsive nanoparticles for anti‐cancer drug delivery. Adv Mater. 2007;19(19):2988.

[7] Torkpur‐Biglarianzadeh M , Salami‐Kalajahi M . Multilayer fluorescent magnetic nanoparticles with dual thermoresponsive and pH‐sensitive polymeric nanolayers as anti‐cancer drug carriers. RSC Adv. 2015;5(38):29653‐29662.

[8] Lo CL , Lin KM , Hsiue GH . Preparation and characterization of intelligent core‐shell nanoparticles based on poly(D,L‐lactide)‐g‐poly(N‐isopropyl‐acrylamide‐co‐methacrylic acid). J Control Release. 2005;104(3):477‐488. 15911047 10.1016/j.jconrel.2005.03.004

[9] Xiang Y , Oo NNL , Lee JP , Li Z , Loh XJ . Recent development of synthetic nonviral systems for sustained gene delivery. Drug Discov Today. 2017;22(9):1318‐1335. 28428056 10.1016/j.drudis.2017.04.001

[10] Kelley EG , Albert JNL , Sullivan MO , Epps TH . Stimuli‐responsive copolymer solution and surface assemblies for biomedical applications. Chem Soc Rev. 2013;42(17):7057‐7071. 23403471 10.1039/c3cs35512h PMC3703495

[11] Uhrich KE , Cannizzaro SM , Langer RS , Shakesheff KM . Polymeric systems for controlled drug release. Chem Rev. 1999;99(11):3181‐3198. 11749514 10.1021/cr940351u

[12] Sundaresan V , Menon JU , Rahimi M , Nguyen KT , Wadajkar AS . Dual‐responsive polymer‐coated iron oxide nanoparticles for drug delivery and imaging applications. Int J Pharm. 2014;466(1–2):1‐7. 24607216 10.1016/j.ijpharm.2014.03.016 PMC4642438

[13] Wong JE , Gaharwar AK , Muller‐Schulte D , Bahadur D , Richtering W . Dual‐stimuli responsive PNiPAM microgel achieved via layer‐by‐layer assembly: magnetic and thermoresponsive. J Colloid Interface Sci. 2008;324(1–2):47‐54. 18514212 10.1016/j.jcis.2008.05.024

[14] Cheng R , Meng FH , Deng C , Klok HA , Zhong ZY . Dual and multi‐stimuli responsive polymeric nanoparticles for programmed site‐specific drug delivery. Biomaterials. 2013;34(14):3647‐3657. 23415642 10.1016/j.biomaterials.2013.01.084

[15] Sun HL , Meng FH , Cheng R , Deng C , Zhong ZY . Reduction and pH dual‐bioresponsive crosslinked polymersomes for efficient intracellular delivery of proteins and potent induction of cancer cell apoptosis. Acta Biomater. 2014;10(5):2159‐2168. 24440420 10.1016/j.actbio.2014.01.010

[16] Townsend DM , Tew KD , Tapiero H . The importance of glutathione in human disease. Biomed Pharmacother. 2003;57(3–4):145‐155. 12818476 10.1016/s0753-3322(03)00043-x PMC6522248

[17] Mura S , Nicolas J , Couvreur P . Stimuli‐responsive nanocarriers for drug delivery. Nat Mater. 2013;12(11):991‐1003. 24150417 10.1038/nmat3776

[18] Xu J , Liu SY . Polymeric nanocarriers possessing thermoresponsive coronas. Soft Matter. 2008;4(9):1745‐1749.

[19] Sammeta SM , Murthy SN . "ChilDrive": a technique of combining regional cutaneous hypothermia with Iontophoresis for the delivery of drugs to synovial fluid. Pharm Res. 2009;26(11):2535‐2540. 19774343 10.1007/s11095-009-9977-0

[20] Wang CX , Yang T , Shuaib A . Effects of minocycline alone and in combination with mild hypothermia in embolic stroke. Brain Res. 2003;963(1–2):327‐329. 12560140 10.1016/s0006-8993(02)04045-3

[21] Levi AD , Casella G , Green BA , et al. Clinical outcomes using modest intravascular hypothermia after acute cervical spinal cord injury. Neurosurgery. 2010;66(4):670‐677. 20190669 10.1227/01.NEU.0000367557.77973.5F

[22] Ramkissoon‐Ganorkar C , Liu F , Baudys M , Kim SW . Modulating insulin‐release profile from pH thermosensitive polymeric beads through polymer molecular weight. J Control Release. 1999;59(3):287‐298. 10332061 10.1016/s0168-3659(99)00006-1

[23] Bae YH , Okano T , Hsu R , Kim SW . Thermosensitive polymers as on‐off switches for drug release. Makromolekulare Chemie‐Rapid Commun. 1987;8(10):481‐485.

[24] Dreher MR , Simnick AJ , Fischer K , et al. Temperature triggered self‐assembly of polypeptides into multivalent spherical micelles. J Am Chem Soc. 2008;130(2):687‐694. 18085778 10.1021/ja0764862 PMC2855373

[25] MacKay JA , Chen MN , McDaniel JR , Liu WG , Simnick AJ , Chilkoti A . Self‐assembling chimeric polypeptide‐doxorubicin conjugate nanoparticles that abolish tumours after a single injection. Nat Mater. 2009;8(12):993‐999. 19898461 10.1038/nmat2569 PMC2862348

[26] Soppimath KS , Tan DCW , Yang YY . pH‐triggered thermally responsive polymer core‐shell nanoparticles for drug delivery. Adv Mater. 2005;17(3):318.

[27] Xu J , Luo SZ , Shi WF , Liu SY . Two‐stage collapse of unimolecular micelles with double thermoresponsive coronas. Langmuir. 2006;22(3):989‐997. 16430258 10.1021/la0522707

[28] Dimitrov I , Trzebicka B , Muller AHE , Dworak A , Tsvetanov CB . Thermosensitive water‐soluble copolymers with doubly responsive reversibly interacting entities. Prog Polym Sci. 2007;32(11):1275‐1343.

[29] Luo SZ , Xu J , Zhu ZY , Wu C , Liu SY . Phase transition behavior of unimolecular micelles with thermoresponsive poly(N‐isopropylacrylamide) coronas. J Phys Chem B. 2006;110(18):9132‐9139. 16671725 10.1021/jp061055b

[30] Topp MDC , Dijkstra PJ , Talsma H , Feijen J . Thermosensitive micelle‐forming block copolymers of poly(ethylene glycol) and poly(N‐isopropylacrylamide). Macromolecules. 1997;30(26):8518‐8520.

[31] Motokawa R , Morishita K , Koizumi S , Nakahira T , Annaka M . Thermosensitive diblock copolymer of poly(N‐isopropylacrylamide) and poly(ethylene glycol) in water: polymer preparation and solution behavior. Macromolecules. 2005;38(13):5748‐5760.

[32] Pietsch C , Mansfeld U , Guerrero‐Sanchez C , et al. Thermo‐induced self‐assembly of responsive poly(DMAEMA‐b‐DEGMA) block copolymers into multi‐ and Unilamellar vesicles. Macromolecules. 2012;45(23):9292‐9302.

[33] Neradovic D , van Nostrum CF , Hennink WE . Thermoresponsive polymeric micelles with controlled instability based on hydrolytically sensitive N‐isopropylacrylamide copolymers. Macromolecules. 2001;34(22):7589‐7591.

[34] Mangold C , Obermeier B , Wurm F , Frey H . From an epoxide monomer toolkit to functional PEG copolymers with adjustable LCST behavior. Macromol Rapid Commun. 2011;32(23):1930‐1934. 21971715 10.1002/marc.201100489

[35] Perrier S . 50th anniversary perspective: RAFT polymerization‐a user guide. Macromolecules. 2017;50(19):7433‐7447.

[36] Semsarilar M , Perrier S . Green' reversible addition‐fragmentation chain‐transfer (RAFT) polymerization. Nat Chem. 2010;2(10):811‐820. 20861895 10.1038/nchem.853

[37] Meyer DE , Chilkoti A . Purification of recombinant proteins by fusion with thermally‐responsive polypeptides. Nat Biotechnol. 1999;17(11):1112‐1115. 10545920 10.1038/15100

[38] Vlieghe P , Lisowski V , Martinez J , Khrestchatisky M . Synthetic therapeutic peptides: science and market. Drug Discov Today. 2010;15(1–2):40‐56. 19879957 10.1016/j.drudis.2009.10.009

[39] Perez CMR , Panitch A , Chmielewski J . A collagen peptide‐based physical hydrogel for cell encapsulation. Macromol Biosci. 2011;11(10):1426‐1431. 21830301 10.1002/mabi.201100230

[40] MacEwan SR , Chilkoti A . Digital switching of local arginine density in a genetically encoded self‐assembled polypeptide nanoparticle controls cellular uptake. Nano Lett. 2012;12(6):3322‐3328. 22625178 10.1021/nl301529p PMC3405287

[41] Shoulders MD , Raines RT . Collagen structure and stability. Annu Rev Biochem Annual Reviews: Palo Alto. 2009;78:929‐958. 19344236 10.1146/annurev.biochem.77.032207.120833 PMC2846778

[42] Sakakibara S , Inouye K , Shudo K , Kishida Y , Kobayashi Y , Prockop DJ . Synthesis of (pro‐hyp‐gly)n of defined molecular‐weights ‐ evidence for stabilization of collagen triple helix by hydroxypyroline. Biochim Biophys Acta. 1973;303(1):198‐202. 4702003 10.1016/0005-2795(73)90164-5

[43] Brodsky B , Ramshaw JAM . The collagen triple‐helix structure. Matrix Biol. 1997;15(8–9):545‐554. 9138287 10.1016/s0945-053x(97)90030-5

[44] Persikov AV , Ramshaw JAM , Brodsky B . Collagen model peptides: sequence dependence of triple‐helix stability. Biopolymers. 2000;55(6):436‐450. 11304671 10.1002/1097-0282(2000)55:6<436::AID-BIP1019>3.0.CO;2-D

[45] Bella J . Collagen structure: new tricks from a very old dog. Biochem J. 2016;473:1001‐1025. 27060106 10.1042/BJ20151169

[46] Li Y , Foss CA , Summerfield DD , et al. Targeting collagen strands by photo‐triggered triple‐helix hybridization. Proc Natl Acad Sci U S A. 2012;109(37):14767‐14772. 22927373 10.1073/pnas.1209721109 PMC3443117

[47] Luo JN , Tong YW . Self‐assembly of collagen‐mimetic peptide Amphiphiles into biofunctional Nanofiber. ACS Nano. 2011;5(10):7739‐7747. 21899363 10.1021/nn202822f

[48] Tanrikulu IC , Forticaux A , Jin S , Raines RT . Peptide tessellation yields micrometre‐scale collagen triple helices. Nat Chem. 2016;8(11):1008‐1014. 27768103 10.1038/nchem.2556 PMC5123832

[49] Sarkar B , O'Leary LER , Hartgerink JD . Self‐assembly of fiber‐forming collagen mimetic peptides controlled by triple‐helical nucleation. J Am Chem Soc. 2014;136(41):14417‐14424. 25494829 10.1021/ja504377s

[50] Rele S , Song YH , Apkarian RP , Qu Z , Conticello VP , Chaikof EL . D‐periodic collagen‐mimetic microfibers. J Am Chem Soc. 2007;129(47):14780‐14787. 17985903 10.1021/ja0758990

[51] O'Leary LER , Fallas JA , Bakota EL , Kang MK , Hartgerink JD . Multi‐hierarchical self‐assembly of a collagen mimetic peptide from triple helix to nanofibre and hydrogel. Nat Chem. 2011;3(10):821‐828. 21941256 10.1038/nchem.1123

[52] Krishna OD , Jha AK , Jia XQ , Kiick KL . Integrin‐mediated adhesion and proliferation of human MSCs elicited by a hydroxyproline‐lacking, collagen‐like peptide. Biomaterials. 2011;32(27):6412‐6424. 21658756 10.1016/j.biomaterials.2011.05.034 PMC3134156

[53] Parmar PA , St‐Pierre JP , Chow LW , et al. Enhanced articular cartilage by human mesenchymal stem cells in enzymatically mediated transiently RGDS‐functionalized collagen mimetic hydrogels. Acta Biomater. 2017;51:75‐88. 28087486 10.1016/j.actbio.2017.01.028 PMC5360098

[54] Meyer DE , Chilkoti A . Quantification of the effects of chain length and concentration on the thermal behavior of elastin‐like polypeptides. Biomacromolecules. 2004;5(3):846‐851. 15132671 10.1021/bm034215n

[55] Koria P , Yagi H , Kitagawa Y , et al. Self‐assembling elastin‐like peptides growth factor chimeric nanoparticles for the treatment of chronic wounds. Proc Natl Acad Sci U S A. 2011;108(3):1034‐1039. 21193639 10.1073/pnas.1009881108 PMC3024670

[56] Bessa PC , Machado R , Nurnberger S , et al. Thermoresponsive self‐assembled elastin‐based nanoparticles for delivery of BMPs. J Control Release. 2010;142(3):312‐318. 19913578 10.1016/j.jconrel.2009.11.003

[57] Wu YQ , MacKay JA , McDaniel JR , Chilkoti A , Clark RL . Fabrication of elastin‐like polypeptide nanoparticles for drug delivery by Electrospraying. Biomacromolecules. 2009;10(1):19‐24. 19072041 10.1021/bm801033f PMC2820340

[58] Hassouneh W , Zhulina EB , Chilkoti A , Rubinstein M . Elastin‐like polypeptide Diblock copolymers self‐assemble into weak micelles. Macromolecules. 2015;48(12):4183‐4195. 27065492 10.1021/acs.macromol.5b00431 PMC4822422

[59] MacEwan SR , Chilkoti A . Elastin‐like polypeptides: biomedical applications of tunable biopolymers. Biopolymers. 2010;94(1):60‐77. 20091871 10.1002/bip.21327

[60] Weitzhandler I , Dzuricky M , Hoffmann I , Quiroz FG , Gradzielski M , Chilkoti A . Micellar self‐assembly of recombinant Resilin−/elastin‐like block Copolypeptides. Biomacromolecules. 2017;18(8):2419‐2426. 28570078 10.1021/acs.biomac.7b00589 PMC6364677

[61] Luo TZ , Kiick KL . Noncovalent modulation of the inverse temperature transition and self‐assembly of elastin‐b‐collagen‐like peptide bioconjugates. J Am Chem Soc. 2015;137(49):15362‐15365. 26633746 10.1021/jacs.5b09941 PMC4930074

[62] Luo TZ , David MA , Dunshee LC , et al. Thermoresponsive elastin‐b‐collagen‐like peptide bioconjugate Nanovesicles for targeted drug delivery to collagen‐containing matrices. Biomacromolecules. 2017;18(8):2539‐2551. 28719196 10.1021/acs.biomac.7b00686 PMC5815509

[63] Wang AY , Foss CA , Leong S , Mo X , Pomper MG , Yu SM . Spatio‐temporal modification of collagen scaffolds mediated by triple helical propensity. Biomacromolecules. 2008;9(7):1755‐1763. 18547103 10.1021/bm701378k PMC3095440

[64] Presolski SI , Hong VP , Finn MG . Copper‐catalyzed Azide‐alkyne click chemistry for bioconjugation. Curr Protocols Chem Biol. 2011;3(4):153‐162. 10.1002/9780470559277.ch110148 PMC3404492 22844652

[65] Pecora R . Dynamic light scattering measurement of nanometer particles in liquids. J Nanopart Res. 2000;2(2):123‐131.

[66] Schneider CA , Rasband WS , Eliceiri KW . NIH image to ImageJ: 25 years of image analysis. Nat Methods. 2012;9(7):671‐675. 22930834 10.1038/nmeth.2089 PMC5554542

[67] Engel J , Bachinger HP . Structure, stability and folding of the collagen triple helix In: Brinckmann J, Notbohm H, Muller PK, eds. Collagen: primer in structure, processing and assembly. Vol 247 Berlin: Springer‐Verlag; 2005:7‐33.

[68] Greenfield NJ . Analysis of the kinetics of folding of proteins and peptides using circular dichroism. Nat Protoc. 2006;1(6):2891‐2899. 17406548 10.1038/nprot.2006.244 PMC2752309

[69] Persikov AV , Ramshaw JAM , Kirkpatrick A , Brodsky B . Amino acid propensities for the collagen triple‐helix. Biochemistry. 2000;39(48):14960‐14967. 11101312 10.1021/bi001560d

[70] MacEwan SR , Chilkoti A . Applications of elastin‐like polypeptides in drug delivery. J Control Release. 2014;190:314‐330. 24979207 10.1016/j.jconrel.2014.06.028 PMC4167344

[71] Perczel A , Hollosi M , Foxman BM , Fasman GD . Conformational‐analysis of pseudocyclic hexapeptides based on quantitative circular‐dichroism (CD), NOE, and X‐RAY data ‐ the pure CD spectra of type‐I and type‐II beta‐turns. J Am Chem Soc. 1991;113(26):9772‐9784.

[72] Shah NK , Ramshaw JAM , Kirkpatrick A , Shah C , Brodsky B . A host‐guest set of triple‐helical peptides: stability of Gly‐X‐Y triplets containing common nonpolar residues. Biochemistry. 1996;35(32):10262‐10268. 8756681 10.1021/bi960046y

[73] Vehring R . Pharmaceutical particle engineering via spray drying. Pharm Res. 2008;25(5):999‐1022. 18040761 10.1007/s11095-007-9475-1 PMC2292490

[74] Urry DW . Physical chemistry of biological free energy transduction as demonstrated by elastic protein‐based polymers. J Phys Chem B. 1997;101(51):11007‐11028.

[75] Reguera J , Urry DW , Parker TM , McPherson DT , Rodriguez‐Cabello JC . Effect of NaCl on the exothermic and endothermic components of the inverse temperature transition of a model elastin‐like polymer. Biomacromolecules. 2007;8(2):354‐358. 17291058 10.1021/bm060936l

[76] Urry DW . Molecular machines ‐ how motion and other functions of living organisms can result from reversible chemical‐changes. Angewandte Chemie‐International Edition in English. 1993;32(6):819‐841.

[77] Urry DW . The change in Gibbs free energy for hydrophobic association ‐ derivation and evaluation by means of inverse temperature transitions. Chemical Phys Lett. 2004;399(1–3):177‐183.

[78] Dai Q , Liu X , Coutts J , Austin L , Huo Q . A one‐step highly sensitive method for DNA detection using dynamic light scattering. J Am Chem Soc. 2008;130(26):8138. 18540598 10.1021/ja801947e

[79] Yanagisawa O , Homma T , Okuwaki T , Shimao D , Takahashi H . Effects of cooling on human skin and skeletal muscle. Eur J Appl Physiol. 2007;100(6):737‐745. 17479279 10.1007/s00421-007-0470-3

[80] Harris JR , Horne RW . Negative staining ‐ a brief assessment of current technical benefits, limitations and future possibilities. Micron. 1994;25(1):5‐13.

[81] Kim KT , Zhu JH , Meeuwissen SA , et al. Polymersome Stomatocytes: controlled shape transformation in polymer vesicles. J Am Chem Soc. 2010;132(36):12522‐12524. 20718470 10.1021/ja104154t

[82] Xu JS , Liu YH , Li YJ , et al. Precise targeting of POLR2A as a therapeutic strategy for human triple negative breast cancer. Nat Nanotechnol. 2019;14(4):388. 30804480 10.1038/s41565-019-0381-6 PMC6449187

[83] Battaglia G , Ryan AJ . Bilayers and interdigitation in block copolymer vesicles. J Am Chem Soc. 2005;127(24):8757‐8764. 15954782 10.1021/ja050742y

[84] Rodriguez‐Cabello JC , Prieto S , Reguera J , Arias FJ , Ribeiro A . Biofunctional design of elastin‐like polymers for advanced applications in nanobiotechnology. J Biomat Science‐Polym Edition. 2007;18(3):269‐286. 10.1163/156856207779996904 17471765

[85] Urry DW . Protein elasticity based on conformations of sequential polypeptides ‐ the biological elastic fiber. J Protein Chem. 1984;3(5–6):403‐436.

[86] Sokolova V , Ludwig AK , Hornung S , et al. Characterisation of exosomes derived from human cells by nanoparticle tracking analysis and scanning electron microscopy. Coll Surfaces B‐Biointer. 2011;87(1):146‐150. 10.1016/j.colsurfb.2011.05.013 21640565

[87] Haney MJ , Klyachko NL , Zhaoa YL , et al. Exosomes as drug delivery vehicles for Parkinson's disease therapy. J Control Release. 2015;207:18‐30. 25836593 10.1016/j.jconrel.2015.03.033 PMC4430381

[88] Bootz A , Vogel V , Schubert D , Kreuter J . Comparison of scanning electron microscopy, dynamic light scattering and analytical ultracentrifugation for the sizing of poly(butyl cyanoacrylate) nanoparticles. Eur J Pharm Biopharm. 2004;57(2):369‐375. 15018998 10.1016/S0939-6411(03)00193-0

[89] Eaton P , Quaresma P , Soares C , et al. A direct comparison of experimental methods to measure dimensions of synthetic nanoparticles. Ultramicroscopy. 2017;182:179‐190. 28692935 10.1016/j.ultramic.2017.07.001

[90] Nishi Y , Uchiyama S , Doi M , et al. Different effects of 4‐hydroxyproline and 4‐fluoroproline on the stability of collagen triple helix. Biochemistry. 2005;44(16):6034‐6042. 15835892 10.1021/bi047887m

