# Supplementary material for: Manipulation of the dually thermoresponsive behavior of peptide‐based vesicles through modification of collagen‐like peptide domains
Source: Bioeng Transl Med. 2019 Oct 16;5(1):e10145. doi: 10.1002/btm2.10145 (PMC6971430; doi:10.1002/btm2.10145)
Supplement: Supplementary file 1 — Appendix S1: Supporting Information [file BTM2-5-e10145-s001.docx]

Manipulation of the Dually Thermoresponsive Behavior of Peptide-Based Vesicles through Modification of Collagen-like Peptide Domains

*Supplementary Information and Data*

Lucas C. Dunshee^†^, Millicent O. Sullivan^†^, Kristi L. Kiick^‡*^

^†^Department of Chemical and Biomolecular Engineering, University of Delaware,
150 Academy Street, Newark, DE 19716 United States of America

^‡^Department of Materials Science and Engineering, University of Delaware,
127 The Green, Newark, DE 19716 United States of America

Lucas C. Dunshee email: ldunshee@udel.edu
Millicent O. Sullivan email: msulliva@udel.edu

Kristi L. Kiick email: kiick@udel.edu

^*^Correspondence should be addressed to K.L.K

Department of Materials Science and Engineering
University of Delaware
102 DuPont Hall
127 The Green
Newark, DE 19716
kiick@udel.edu
+1-302-831-0201 (p)
+1-302-831-4545 (f)

*Section 1: Supplementary Information*

*Section 1.1: Experimental Procedures for Peptide Synthesis and Purification*

All residues of each peptide were incorporated automatically (with the exception of the N-terminal azide of the CLPs) on a Tribute® automatic peptide synthesizer (Gyros Protein Technologies, Tucson, AZ) instrument with 1.5 hour long double couplings for every residue and with every coupling taking place in the presence of 0.4 M 4-methylmorpholine and a 6-fold excess of amino acid and 5.9 fold HBTU molar excess over the 0.2 mmol synthesis scale of the resin. Between couplings the resin was washed five times with excess dimethylformamide (DMF). The deprotection of the Fmoc from each residue was performed on the automatic peptide synthesizer in 20% v/v piperidine in DMF and was carried out two times for ten minutes per deprotection step. After deprotection the resin was washed five times with excess DMF, after which the subsequent coupling was then performed. Due to the light and temperature sensitive nature of 4-azidobutyric acid, this compound was double coupled to the N-terminus of all CLP peptides manually for 1.5 hours with a 15 mL (DMF) reaction volume in a 25 mL peptide synthesis reaction vessel with at 15 fold molar excess of 4-azidobutanoic acid and diisopropylethylamine (DIPEA), and a 14.5 fold molar excess of the HBTU over the 0.2 mmol scale that was used for the resin.

All peptides were cleaved in the same manner with 15 mL of a standard cleavage cocktail composed of 95 % v/v trifluoroacetic acid, 2.5% v/v triisopropylsilane, and 2.5% v/v HPLC-grade water. The cleavage reaction was carried out in a 25 mL peptide synthesis reaction vessel that was shaken for three hours on a Burrell Model 75 wrist action shaker (Burrell Scientific LLC., Pittsburgh, Pennsylvania, United States). The cleavage solution was then separated from the resin via the peptide synthesis reaction vessel into a 50 mL conical tube and partially evaporated to approximately 10 mL using nitrogen gas. The concentrated peptide cleavage solution was then precipitated into 35 mL of pre-chilled (-20°C) anhydrous diethyl ether. The precipitate was pelleted in a 50 mL conical tube via centrifugation at 4000 rpm for seven minutes. The ether was decanted from the pelleted precipitate before adding an additional volume of fresh diethyl ether to wash the precipitate. After a second centrifugation at identical conditions, the diethyl ether was then decanted and the wet peptide cake was allowed to dry at room temperature overnight before subsequent dissolution in water and lyophilization that took place for 60 hours.

After cleavage and lyophilization, crude peptides were purified using reverse phase high performance liquid chromatography (RP-HPLC), with a Viva C18 (150 mm x 30 mm, 5 μm particle size, 30 nm pore size) column from Restek (Lancaster, PA) on a Prominence chromatography HPLC unit (Shimadzu Inc., Columbia, MD). The quality and purity of collected peptide HPLC fractions were checked and directly analyzed via an ultra-performance liquid chromatographic unit in line with an electrospray ionization Xevo G2-S QTof mass spectrometer (denoted UPLC-MS) (Waters Corporation, Milford, MA). Total ion chromatograms and mass spectra are given in supplementary Figures S1-S6. High purity for each peptide was indicated by UPLC-MS analysis (Figures S1 and S6a). After verification of mass and purity, the peptides were lyophilized for 60 hours after which time the peptides were used for further characterization or conjugation.

*Section 1.2: Experimental Procedures for ELP-CLP Synthesis and Purification*

ELP-CLP conjugates were synthesized by an adapted version of the copper (I) mediated azide-alkyne cycloaddition reaction that has been summarized by *Presolski etal*.^1^ Briefly, 6 μmoles of CLP and 3 μmoles of ELP were weighed and transferred to a 10 mL scintillation vial with an appropriately sized stir bar. Stock solutions of 200 mM Cu(II)sulfate, 300 mM THPTA ligand, 2M (+)-sodium L-ascorbate, and 2M aminoguanidine hydrochloride were prepared in HPLC-grade water. For copper-ligand coordination, 30 μL of stock Cu(II)sulfate and 117 μL of THPTA ligand were premixed in a 250 μL microcentrifuge tube. The entire ligand/Cu(II)sulfate solution was then added to the 10 mL scintillation vial (containing the ELP and CLP) along with appropriate amounts of (+)-sodium L-ascorbate, and aminoguanidine hydrochloride to give the following final concentration of each reagent: 6 mM CLP, 3 mM ELP, 6 mM Cu(II)sulfate, 35 mM THPTA, 400 mM (+)-sodium L-ascorbate, and 300 mM aminoguanidine hydrochloride with all components dissolved in 70 % HPLC-grade water v/v and 30 % v/v DMSO. The reaction was carried out for approximately 1 hour with constant stirring at a temperature corresponding to that of the relevant CLP melting temperatures as determined by CD spectroscopy. It should be noted that aminoguanidine hydrochloride was only included for the F6-GFOGER conjugate sequence due to presence of the arginine residue that could limit the reaction through side reactions between dehydroascorbate and the guanidine side chain.^1^

After completion of the 1 hour reaction, all conjugate reaction solutions were injected directly onto an RP-HPLC column for purification. Similar to the CLPs and ELPs, the mass spectra of each of the collected HPLC fractions of each conjugate were analyzed via UPLC-MS. Total ion chromatograms and mass spectra are given in supplementary Figures S7-S11.

*Section 1.3: Experimental Parameters for Fourier Transform Infrared Spectroscopy (FTIR)*

A Tensor 27 Bruker (Bruker, Billerica, MA) Fourier Transform Infrared (FTIR) spectrophotometer coupled with a diamond lens Attenuated Total Reflectance (ATR) unit was utilized for generation of infrared absorbance spectra of all peptides and conjugates. For these experiments, previously lyophilized dry peptide or conjugate powder was deposited directly onto the diamond lens and then pressed with an equipped flat tipped anvil. Spectra were generated with 256 scans at a data interval of 0.5 cm^-1^ and a resolution of 2 cm^-1^. This technique was utilized to confirm the presence of azide in CLP peptides (by observing the 2169 cm^-1^ to 2080 cm^-1^ peak region) as well as the disappearance of the azide functionality of the ELP-CLP conjugates.^2^ As expected, the 2100 cm^-1^ peak (indicative of azide functionality) was only found to be present on the azide-functionalized CLPs and not the ELP or any of the purified conjugate sequences. Since the CLP was in excess relative to the ELP in the CuAAC reaction, the lack of any azide functionality present in the conjugate indicated that purification of the conjugate completely removed unconjugated CLP from the final pure conjugate product (Figure S12).

*Section 1.4: CD Experimental Procedures and Calculations*

Characterization of triple helices was performed via CD spectroscopy of all peptides and conjugates. A Jasco 810 CD spectropolarimeter (Jasco, Easton, MD) was used for all CD experiments along with a 0.2 cm path length cuvette. CLP and ELP peptides were studied at a concentration of 0.35 mM in HPLC grade water (pH 6.5 at 4°C) for all experiments (wavelength and melting curve) and all conjugates were studied at a concentration of 0.10 mM for wavelength scans and a concentration of 0.35 mM for melting curve experiments, each in HPLC grade water (pH 6.5 at 4°C). Each complete experiment consisted of first recording a wavelength scan at 4°C from 250 nm to 218 nm (205 nm for conjugate; the differences in the lower wavelength achievable resulted from concentration-induced changes in dynode voltages at the lower wavelengths) to check for triple helix formation. This 4°C wavelength scan was followed by a variable temperature analysis that continuously monitored the canonical triple helix maxima at 225 nm over the course of heating from 4°C to 80°C with a heating rate of 10°C/hour. At the end of the acquisition of the melting curve, a second wavelength scan at 80°C was performed from 250 nm to 218 nm (205 nm for the conjugate). All wavelength scan data (at 4°C and 80°C) are provided in Figures S13, S14a, and S15 (CLP peptide, ELP peptide, and conjugate scans, respectively, with the exception of the GPP10 CLP and F6-GPP10 conjguate). All wavelength scans were acquired as an average of three separate measurements (accumulation = 3) with data collection at 1 nm intervals, a 1nm bandwidth, a 4 second response time, and a scanning rate of 10 nm/min. Prior to all 4°C wavelength scans, the sample was incubated at 4°C overnight and prior to all 80°C wavelength scans the samples were melted out during the melting experiments followed by a 0.5 hour incubation at 80°C. It should briefly be mentioned that slightly different procedures were utilized for acquiring wavelength scans of F6-GPP10 relative to the other ELP-CLP conjugates. The caption to Figure S16 states the detailed procedures that were used. All other measurement parameters were identical to the other conjugates.

All varied temperature (e.g., melting curve) experiments were performed with a 0.2°C data pitch, a 1 nm bandwidth, a 2 second response time, and a heating rate of 10°C/hour. The minima of a first derivative of a Boltzmann function fitting to the CLP and ELP-CLP conjugate melting curve data was defined as the melting temperature for each peptide/conjugate. All data were outputted from the Jasco spectropolarimeter as ellipticity in millidegrees versus temperature or wavelength. The Boltzmann function fitting of the melting curve data as well as the first derivative calculation were completed using Origin 2017 software (Originlab Corporation, Northampton, MA). The ellipticity data were subsequently converted to the mean residue ellipticity [θ_MRE_] as shown in equation 1.1 below:

$$\left[ \theta_{MRE} \right]\left( degM^{-1}m^{-1} \right)= \frac{\left[ \theta\right]\left( mdeg \right)}{l \left( mm \right)*c \left( M \right)*N} (1.1)$$

in which [θ] is the outputted ellipticity in millidegrees (mdeg), l is the path length of the cuvette in millimeters, c is the concentration of the peptide in molar units, and N is the number of residues in a single peptide sequence. The ELP melting curve scan is provided in Figure S14b and all CLP melting curves are provided in Figure S17. It should briefly be noted that there is no significant positive contribution at 225 nm from the ELP at 4°C and only a minor negative ellipticity at this wavelength at 80°C (Figure S14a). Additionally, the ellipticity change of the ELP at 225 nm through the course of heating from 4°C to 80°C is linear and is not representative of a secondary structure transition (Figure S14b).^3^

For the discussion regarding the mechanism of the CLP mediated change in the inverse transition temperature (T_t_) of ELP-CLP conjugates, the degree of helicity (fraction of triple helix folded, F) was calculated by the equation given by Engel and Bächinger,^4^ which is as follows:

$$F= \frac{\left[ \theta_{MRE} \right]-[\theta_{M}]}{\left[ \theta_{T} \right]-[\theta_{M}]}*100 (1.2)$$

where [θ_MRE_] is the measured (and converted) ellipticity, [θ_M_] is the mean residue ellipticity of completely melted CLP (in monomeric form), and [θ_T_] is the mean residue ellipticity of completely folded CLP (in triple helical form). In this work, [θ_M_] is the average of the last five data points of the Boltzmann fit of each melting curve scan (corresponding to 80°C), and [θ_T_] is the average of the first five data points of the Boltzmann fit of each melting curve scan (corresponding to 4°C).

*Section 1.5: Details of Sample Preparation for Transmission Electron Microscopy*

After dispensing 18 µL of 1 mg/mL solution of vesicles into the well of a Nunc® MicroWell® Mini Tray, a pre-ionized carbon coated copper grid was taken and rested on the surface of the vesicle containing droplet for one minute. During this time, 18 μL of 1 wt % phosphotungstic acid (PTA) in water was pipetted into three separate adjacent wells for the F6-GFOGER and F6-GPO7 conjugate bilayer observation or just one well for the F6-GPP10 and F6-GPO7 conjugate for general particle morphology. After one minute, the grid was quickly transferred to each of the one (or three for F6-GFOGER and F6-GPO7) PTA stain droplets sequentially, with the grid resting on the surface of each droplet only for a few seconds. After resting briefly on the only (or third) PTA stain droplet, the grid was blotted dry from the top (opposite to the side resting on the various droplets) using Whatman grade 1 filter paper. Once no more liquid could be wicked away the grid was deemed dry and ready for immediate TEM analysis.

*Section 1.6: Discussion Regarding the Induction of F6-GFOGER Conjugates*

After the determination that the F6-GPO7 conjugate possesses a dissociation temperature and behavior quite similar to the T_t_ phenomena in ELPs (e.g., a fast-onset and fully reversible transition), we postulated that the T_t_ of both the F6-GPO6 and F6-GFOGER conjugate could be amended by other means in addition to CLP folding. In the ELP literature, it is well known that that the T_t_ can be lowered (and thus coacervation induced) through the addition of salt (e.g. NaCl).^5,6^ The effect of NaCl is magnified when the ELP contains a charged residue such as glutamic acid or arginine,^7^ motivating analysis of the impact of NaCl (100 mM) on the assembly of F6-GFOGER. To assess the change in T_t_ imparted by the addition of NaCl, the D_h_ distributions at 80°C, 65°C, 50°C, 35°C, 30°C, and 25°C were monitored in a 1 mg/mL solution of F6-GFOGER in 100 mM aqueous NaCl (Figure S19a). As was the case for the F6-GFOGER conjugate in pure water (Figure 2b), only small D_h_ values were observed at temperatures of 80°C, 65°C, and 50°C, suggesting a lack of vesicle formation. However, similar to the F6-GPO7 and F6-GPP10 conjugates that possessed an intermediate diameter when cooled to their T_m_ of 50°C, cooling the F6-GFOGER salt solution to temperatures near that of its T_m_, inducted formation of vesicles based upon the significant jump in D_h_ and the marked increase in the autocorrelation function delay time (Figure S20a, with replicate distribution data in Figure S20b). Further cooling to 25°C resulted in a moderate increase in diameter (to approximately 100 nm) as the vesicles likely continued to self-assemble in a manner that is concurrent with the folding of the CLP domain into a triple helix. These data suggest the successful reduction in the T_t_ of the F6-GFOGER conjugate by the addition of salt, which is consistent with trends in ELP literature.^5-7^

To determine the morphology of the observed vesicles, TEM analysis was performed at 25°C. Two representative images (Figure 19b,c) show that the F6-GFOGER self-assembly resulted in spherical vesicles with a well-defined bilayer that was measured to have a thickness of 13.3 ± 3.3 nm, and an average vesicle diameter of 52 ± 10 nm. The dimensions of the F6-GFOGER vesicle bilayer and diameter are similar to that of the F6-GPO7 vesicles owing to the similarity in the length of the CLP domains in the two conjugates. See the following Supplementary Information section 1.7 for more details regarding the bilayer calculation.

*Section 1.7: Discussion Regarding Bilayer Thickness*

The measured dimensions of the measured ELP-CLP bilayers are in reasonable agreement with expected for the molecular length scales of these conjugates given that a (Gly-Pro-Hyp) repeat has been shown to have a unit length of approximately 0.9 nm,^8,9^ and that a single ELP β-spiral revolution comprised of six pentamers has a unit length of approximately 1 nm.^5,10,11^ Based on these dimensions, a single trimer of the F6-GPO7 conjugate should exhibit a length of 7.3 nm and a bilayer of approximately 14.1 ± 0.5 nm in thickness.

*Section 1.8: Experimental Parameters and Discussion Regarding F6-GPP10 Aggregation and Kinetics*

To assess the thermal responsiveness of these aggregates, turbidity measurements were performed with heating or cooling temperature gradients applied over 22 minutes. Directly after particle formation, a cuvette-sized stir bar was added to the 1 mg/mL F6-GPP10 samples and the cuvette was inserted into to a Cary 60 UV-Vis spectrophotometer equipped with a temperature and magnetic stirring controller. The heating turbidity experiment was performed by heating the cuvette solution from 25°C to 80°C with a 2.5°C/min heating rate; the solution was continuously monitored for turbidity using a 600 nm laser with constant stirring rate of 400 rpm. The data for the heating experiment are given in Figure 5a of the main text but are also provided as the black diamond data in Figure S26a for convenience. Directly following the heating analysis, the solution was then cooled from 80°C to 25°C with a 2.5°C/min cooling rate. This data is also provided in Figure S26a (blue diamonds) and shows that the re-cooling of the F6-GPP10 conjugate did not result in re-aggregation within the ca. 20-min duration of the cooling. Immediately following this cooling, the turbidity of the solution was monitored without stirring at 25°C over the course of ~6 hours (Figure S26b). Almost immediately following the cooling in Figure S26a, re-aggregation began to occur and aggregates began to either increase in number or size (or both) as evidence by the increasing turbidity, until approximately 3 hours in to the 6 hour experiment. After that time, the aggregates began to settle, presumably due to their increased size (Figure S26b). Throughout the important regimes in this aggregation and settling process, aliquots were taken from the solution for subsequent TEM analysis in order to better understand the morphological characteristics of the aggregates. The times of these aliquots are demarked in Figure S26b with black arrows and the TEM images for the 45 minute, 200 minute, and 9 hour time points are shown in Figure S26c), d) and e), respectively. The TEM images of these aggregates over the course of time show small discrete (non-spherical) particles forming first, followed by clustering/flocculation of these particles; finally, large unstructured aggregates formed well after the turbidity study ended. The morphologies were consistent with the observed turbidity data in Figure 26b), given that particles with small sizes and low number densities would not scatter as much light as large aggregates would, and eventually, settling would produce a decrease in overall turbidity.

The aggregation profile of F6-GPP10 may result from the differences in the hydrophobicity between the (GPP) units of the F6-GPP10 and the (GPO) units of the F6-GPO7 and F6-GFOGER. In these samples, increased van der Waals attractive forces may be driving a flocculation process.^12^ Alternatively, the chain-length and volume fraction of CLP domain could be inducing the formation of non-discrete aggregates such as lamellae^13^ or bilayer rod-transition intermediates.^14^

*Section 2: Supplementary Data*


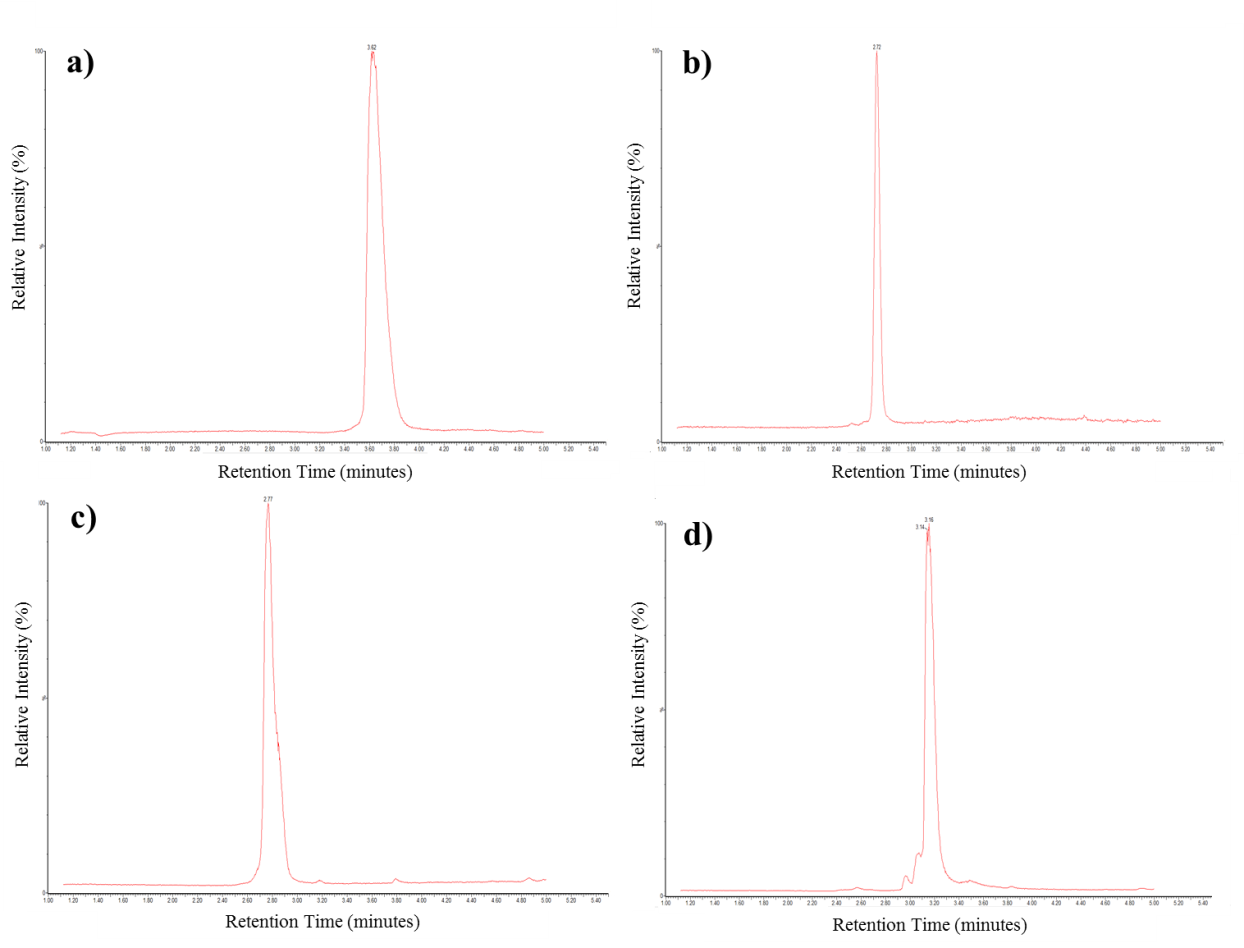


**Figure S1.** Ultra-performance liquid chromatography total ion chromatograms of RP-HPLC purified peptides; a) azide-(GPO)6GG, b) azide-(GPO)3GFOGER(GPO)3GG, c) azide-(GPO)7GG, d) azide-(GPP)10GG. Integrations of the chromatograms revealed that each CLP comprised greater than 95% of the total chromatogram, indicating each CLP was highly pure.


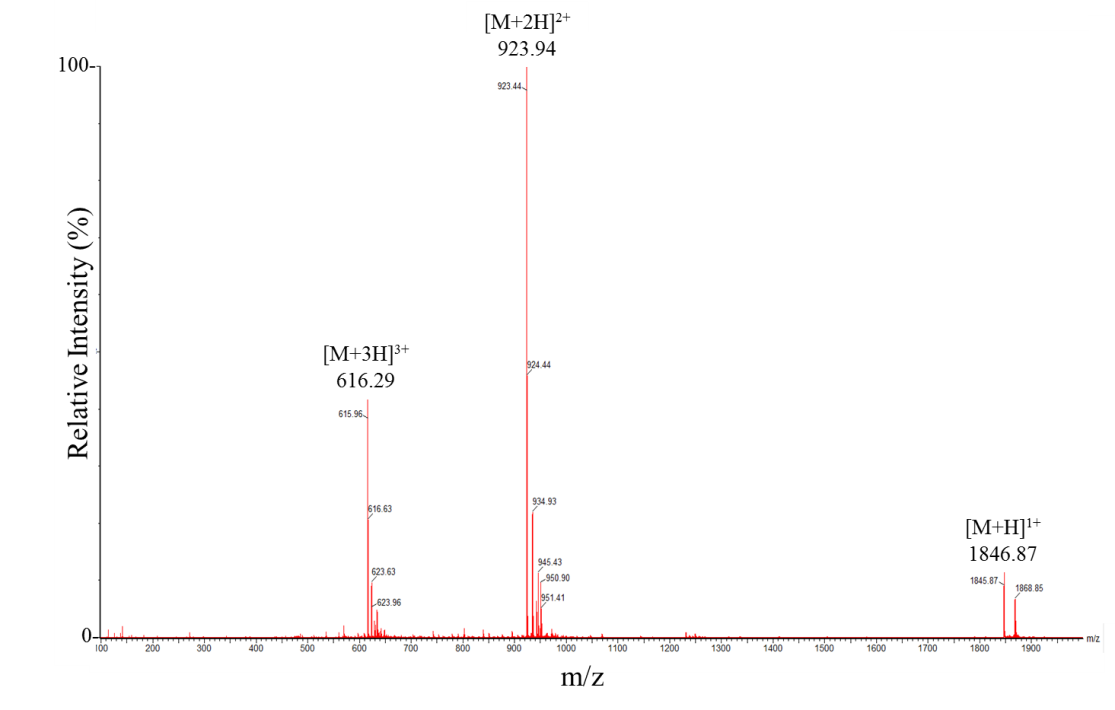


**Figure S2.** Electrospray ionization mass spectrometry of RP-HPLC purified azide-(GPO)6GG. Expected exact mass = 1844.84 Da with the observed adducts m/z = 616.29 [(M+3H)^3+^, calculated = 615.95], m/z = 923.94 [(M+2H)^2+^, calculated = 923.43], and m/z = 1846.87 [(M+H)^1+^, calculated = 1845.85].


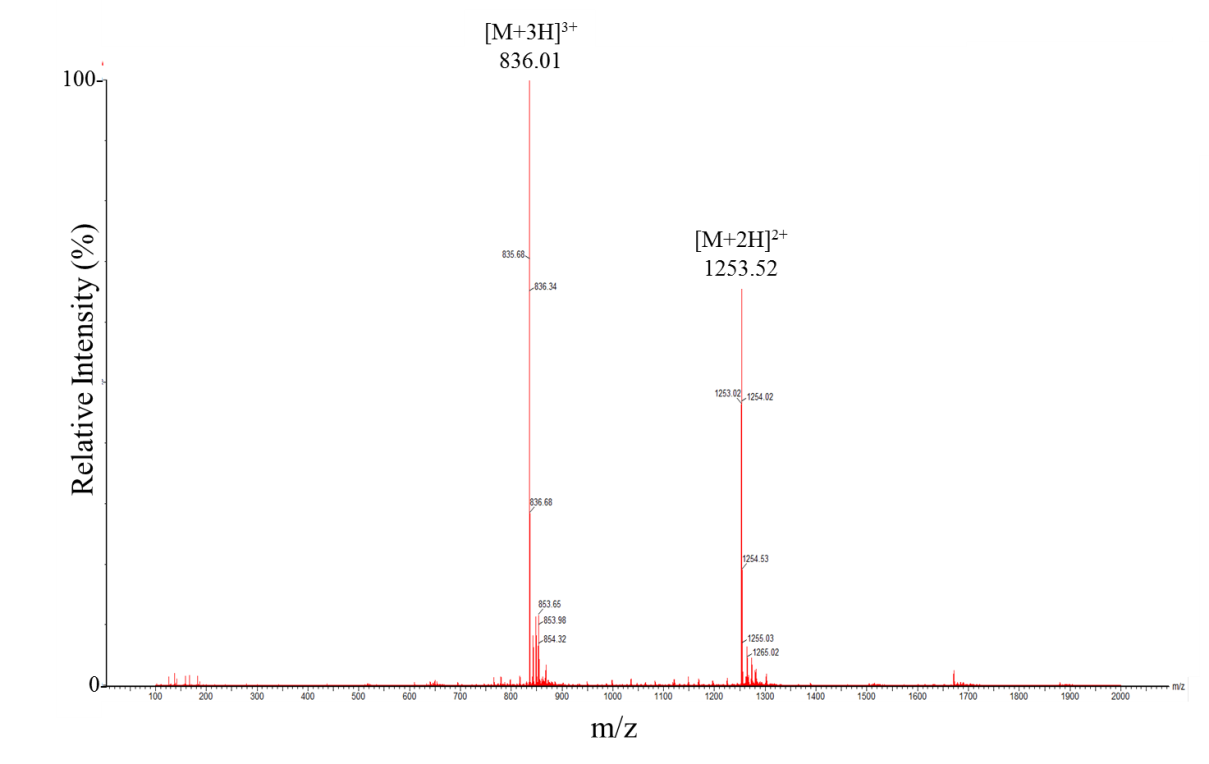


**Figure S3.** Electrospray ionization mass spectrometry of RP-HPLC purified azide-(GPO)3GFOGER(GPO)3GG. Expected exact mass = 2504.15 Da with the observed adducts m/z = 836.01 [(M+3H)^3+^, calculated = 835.7] and m/z = 1253.52 [(M+2H)^2+^, calculated = 1253.08].


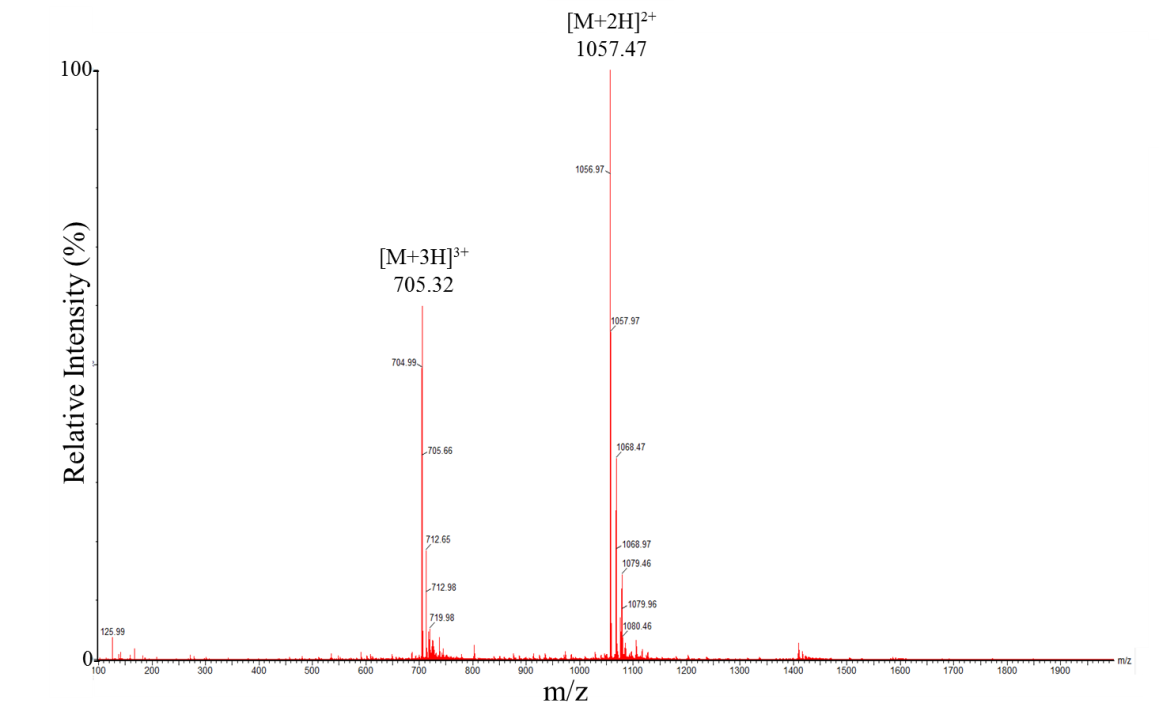


**Figure S4.** Electrospray ionization mass spectrometry of RP-HPLC purified azide-(GPO)7GG. Expected exact mass = 2111.96 Da with the observed adducts m/z = 705.32 [(M+3H)^3+^, calculated = 704.99] and m/z = 1057.47 [(M+2H)^2+^, calculated = 1056.98].


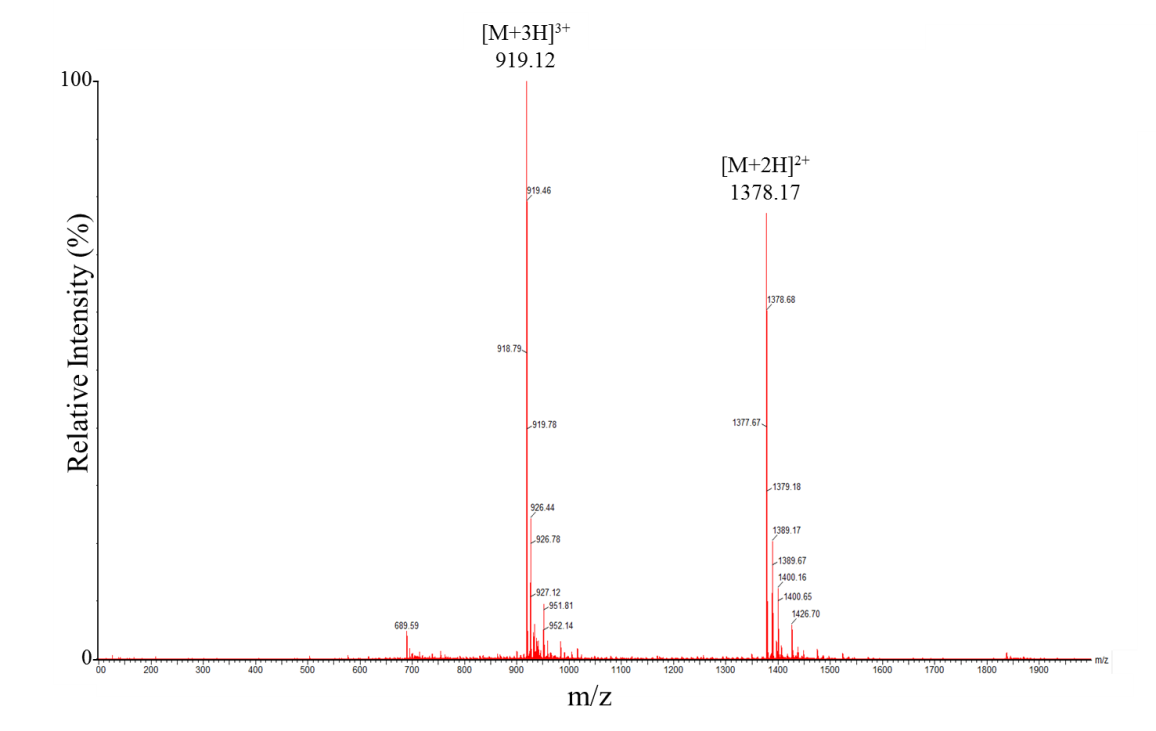


**Figure S5.** Electrospray ionization mass spectrometry of RP-HPLC purified azide-(GPP)10GG. Expected exact mass = 2753.38 Da with the observed adducts m/z = 919.12 [(M+3H)^3+^, calculated = 918.8] and m/z = 1378.17 [(M+2H)^2+^, calculated = 1377.69].


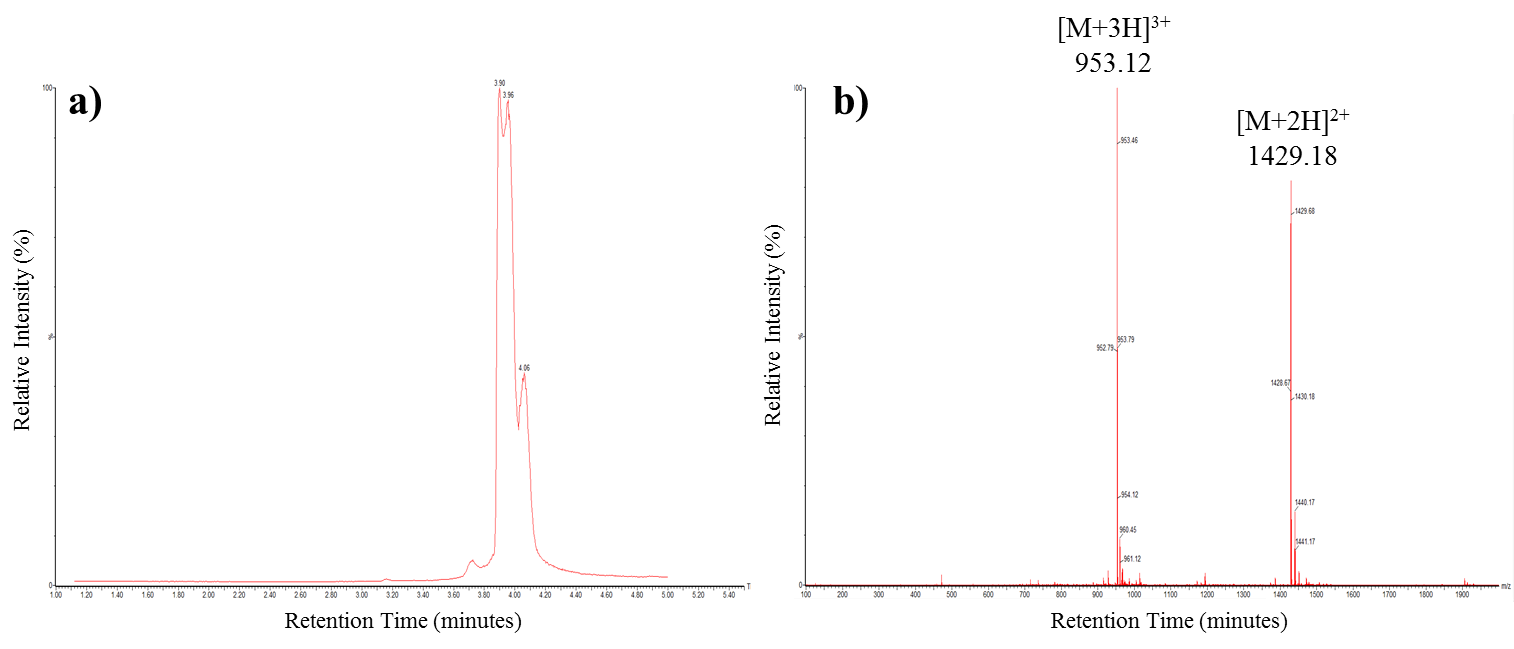


**Figure S6.** a) Ultra performance liquid chromatography total ion chromatograms of RP-HPLC (VPGFG)6G’; while there are minor peaks eluting at nearly the same time, it is reasoned that the peptide is aggregating to a small degree and therefore eluting at slightly differing times. This is reasoned to be the case given the later eluting peaks also possess mass spectra representative of (VPGFG)6G’. Integration of the chromatogram revealed that ELP comprised greater than 90% of the total chromatogram, indicating that the ELP was highly pure. b) Electrospray ionization mass spectrometry of RP-HPLC purified (VPGFG)6G’; expected exact mass = 2855.45 Da with the observed adducts m/z = 953.12 [(M+3H)^3+^, calculated = 952.82] and m/z = 1429.18 [(M+2H)^2+^, calculated = 1428.73].


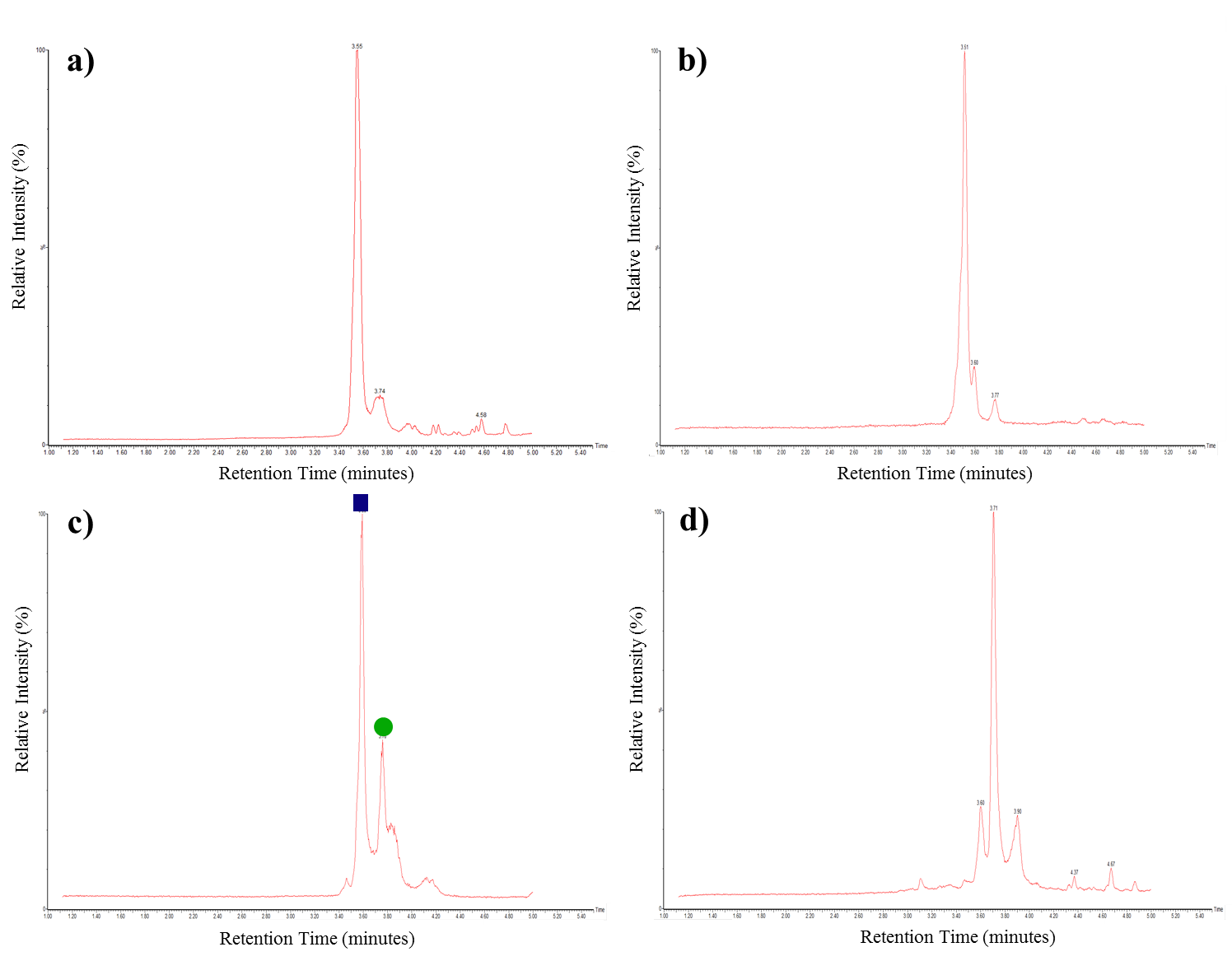


**Figure S7**. Ultra-performance liquid chromatography total ion chromatograms of RP-HPLC purified ELP-CLP conjugates; a) (VPGFG)6-(GPO)6GG, b) (VPGFG)6-(GPO)3GFOGER(GPO)3GG, c) (VPGFG)6-(GPO)7GG, d) (VPGFG)6-(GPP)10GG. Integrations of all of the peaks in each of the chromatograms revealed that each conjugate comprised greater than 95% of the total chromatogram, indicating that the conjugate was highly pure. Note that the peaks that eluted slightly later than the main peak for each of the conjugates possessed the same mass spectra as the main peak, indicating that triple helical dimers and trimers were occurring due to slightly folded complexes that were forming prior to injection on the column. Thus, the purity of each conjugate is determined from the sum of the peaks that possess the correct conjugate mass. An example of this is given in Figure S10 below where the mass spectra of the blue square and the green circle are compared directly for the (VPGFG)6-(GPO)7GG conjugate shown in c) above.


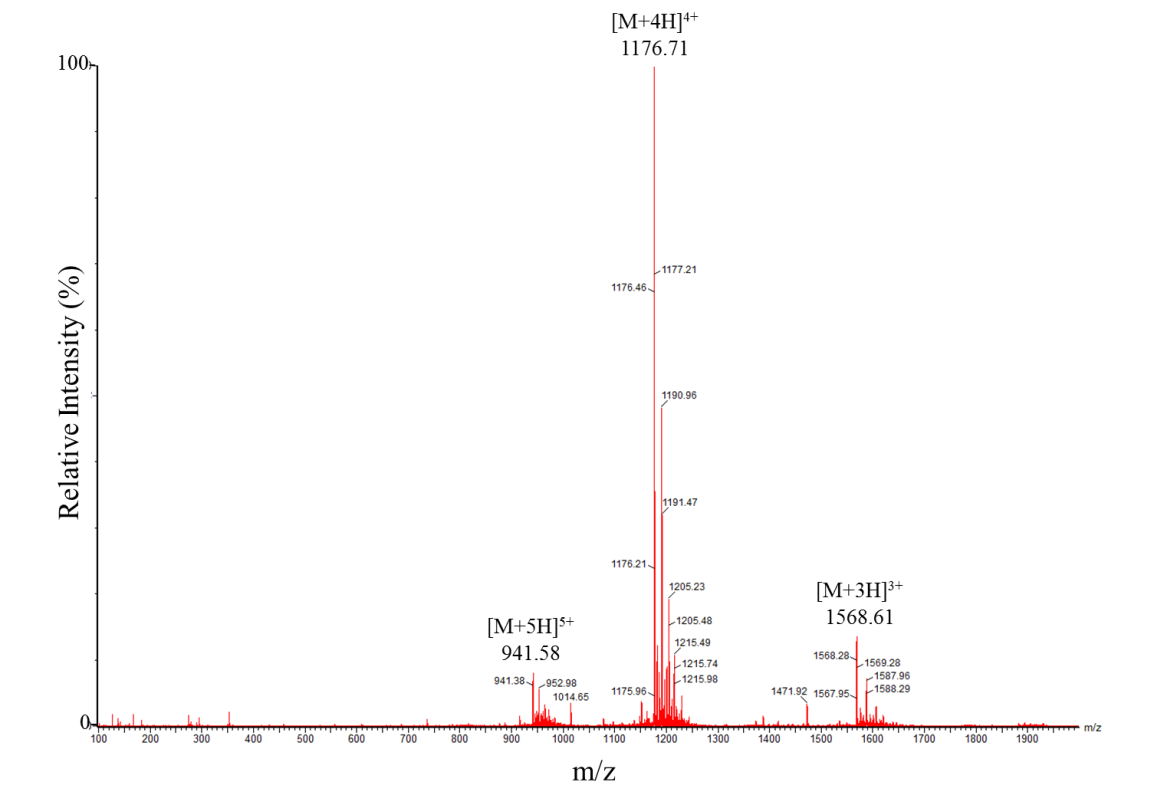


**Figure S8**. Electrospray ionization mass spectrometry of RP-HPLC purified (VPGFG)6-(GPO)6GG. Expected exact mass = 4700.30 Da with the observed adducts m/z = 941.58 [(M+5H)^5+^, calculated = 941.06], m/z = 1176.71 [(M+4H)^4+^, calculated = 1176.08], and m/z = 1568.61 [(M+3H)^3+^, calculated = 1567.77] .


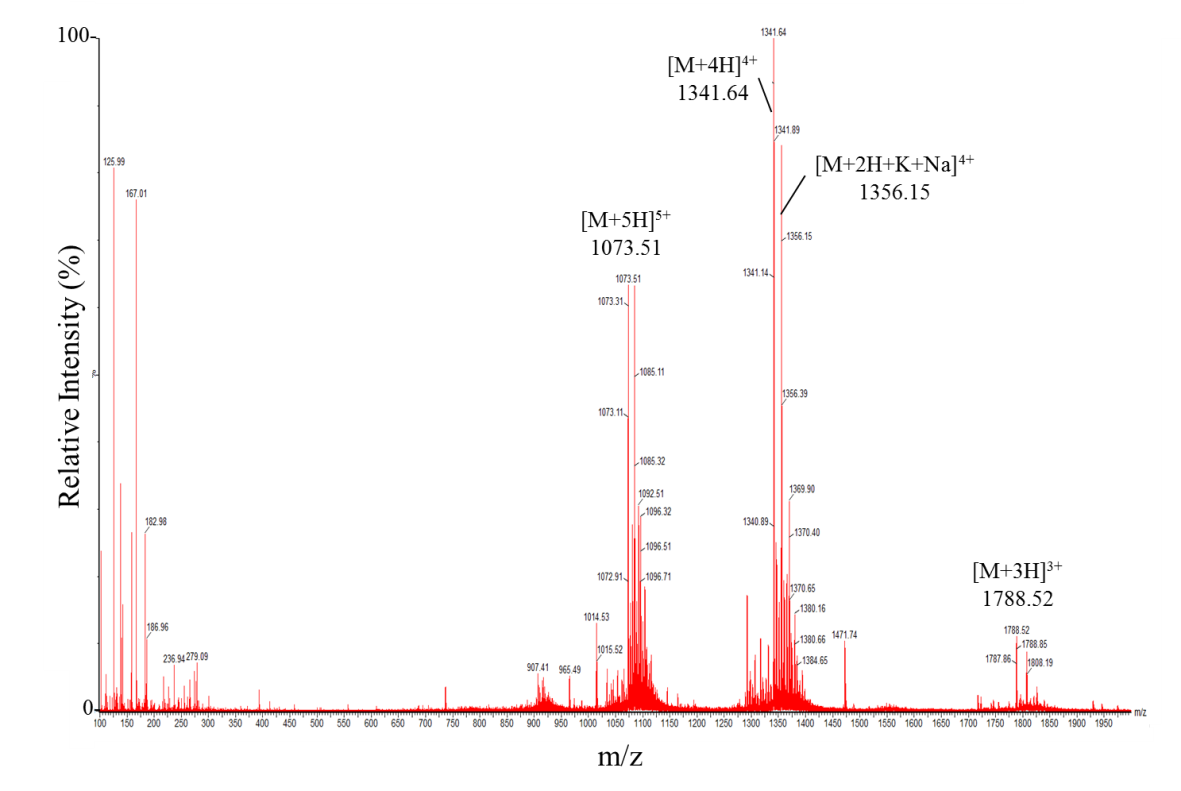


**Figure S9**. Electrospray ionization mass spectrometry of RP-HPLC purified (VPGFG)6-(GPO)3GFOGER(GPO)3GG. Expected exact mass = 5359.6 Da with the observed adducts m/z = 1073.51 [(M+5H)^5+^, calculated = 1072.93], m/z = 1341.64 [(M+4H)^4+^, calculated = 1340.91], m/z = 1356.15 [(M+2H+K+Na)^4+^, calculated = 1355.89], and m/z = 1788.52 [(M+3H)^3+^, calculated = 1787.54] .


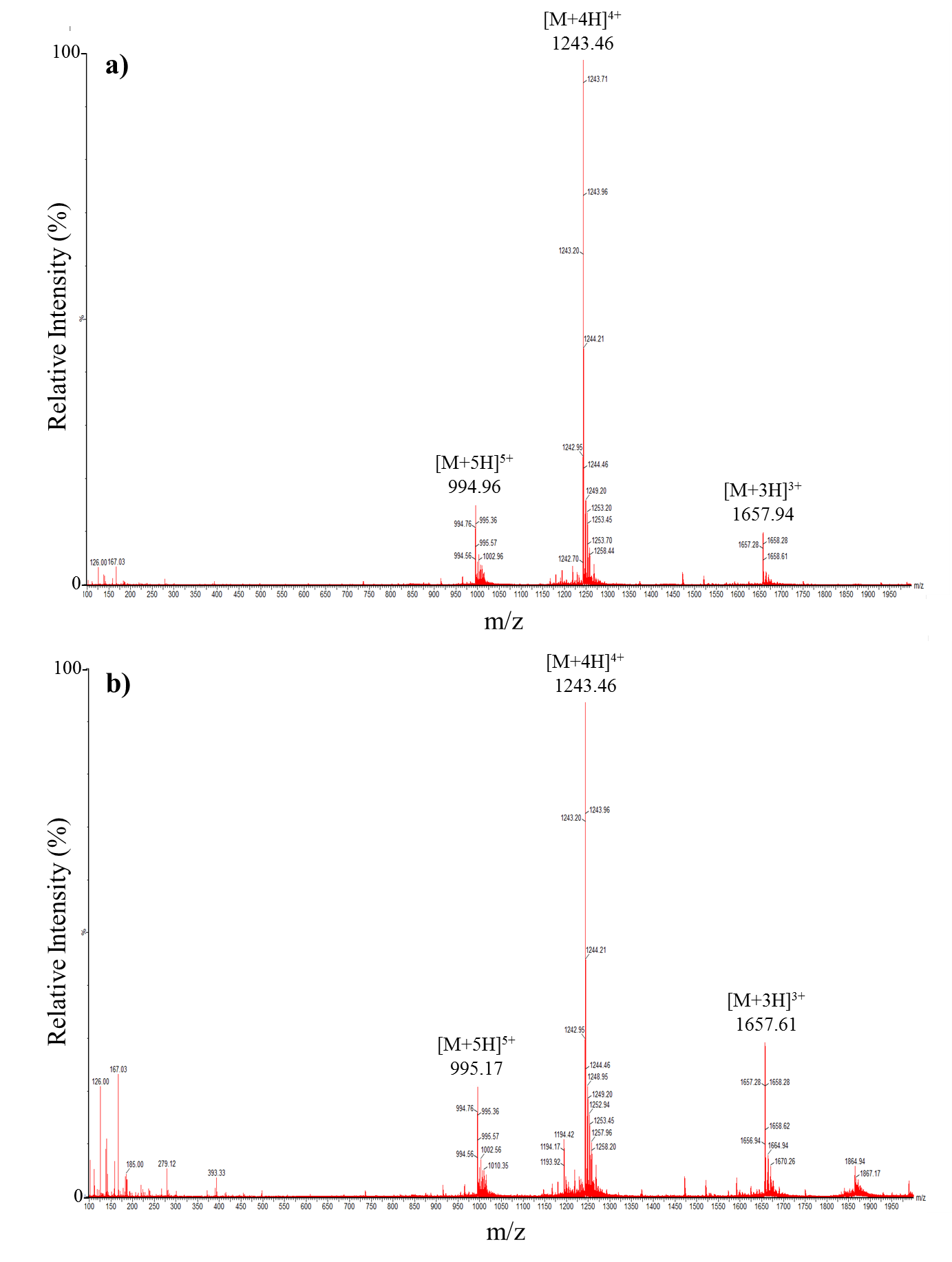


**Figure S10**. Electrospray ionization mass spectrometry of RP-HPLC purified (VPGFG)_6_-(GPO)_7_GG; a) mass spectra of chromatogram peak in Figure S7c marked with blue square, b) mass spectra of chromatogram peak in Figure S7c marked with green circle. Expected exact mass = 4967.43 Da with the observed adducts m/z = 994.96 [(M+5H)^5+^, calculated = 994.49], m/z = 1243.46 [(M+4H)^4+^, calculated = 1242.86], and m/z = 1657.94 [(M+3H)^3+^, calculated = 1656.82]. The small intensity, but large valued m/z in b) (~1800 Da) was determined to be from dimerized conjugate.


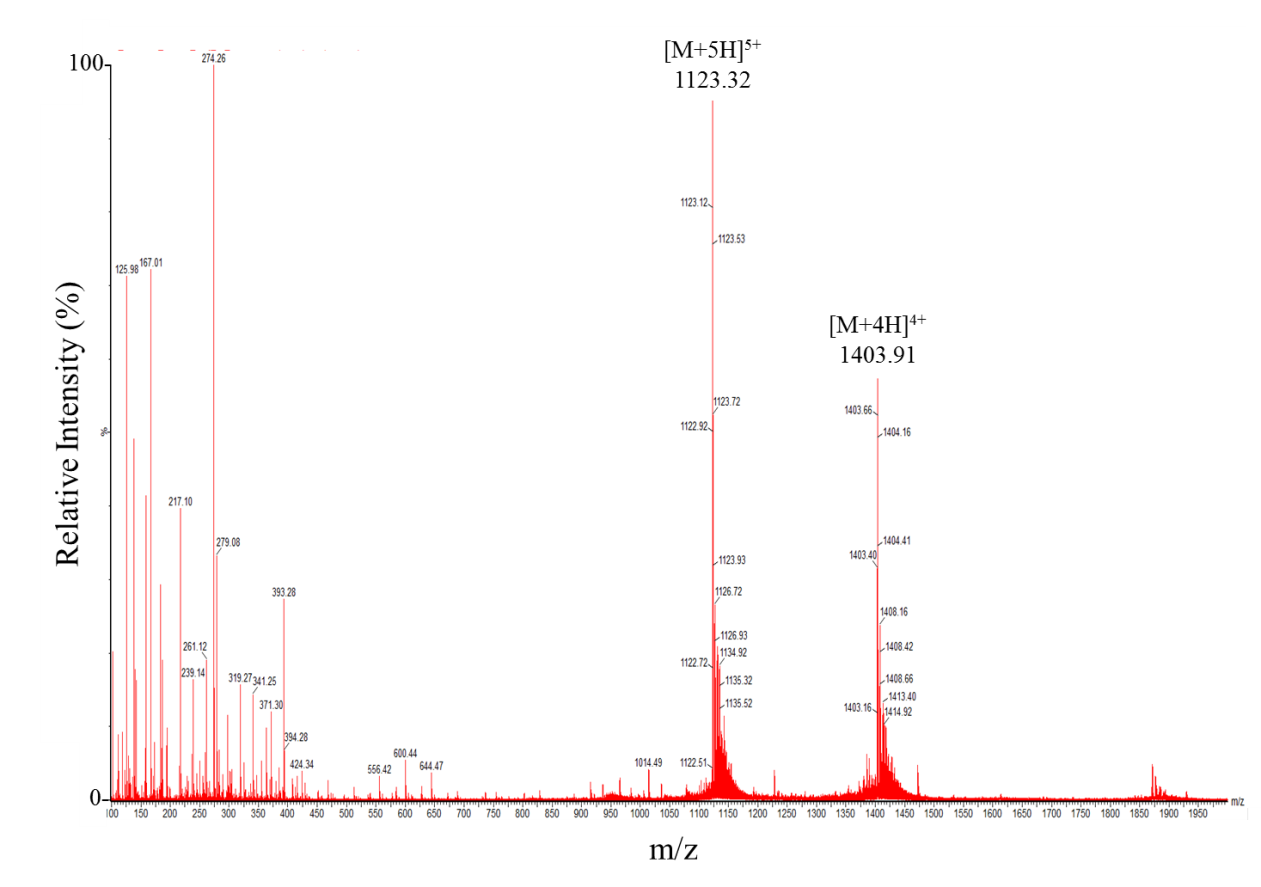


**Figure S11**. Electrospray ionization mass spectrometry of RP-HPLC purified (VPGFG)6-(GPP)10GG. Expected exact mass = 5608.9 Da with the observed adducts m/z = 1123.32 [(M+5H)^5+^, calculated = 1122.78] and m/z = 1403.91 [(M+4H)^4+^, calculated = 1403.23].


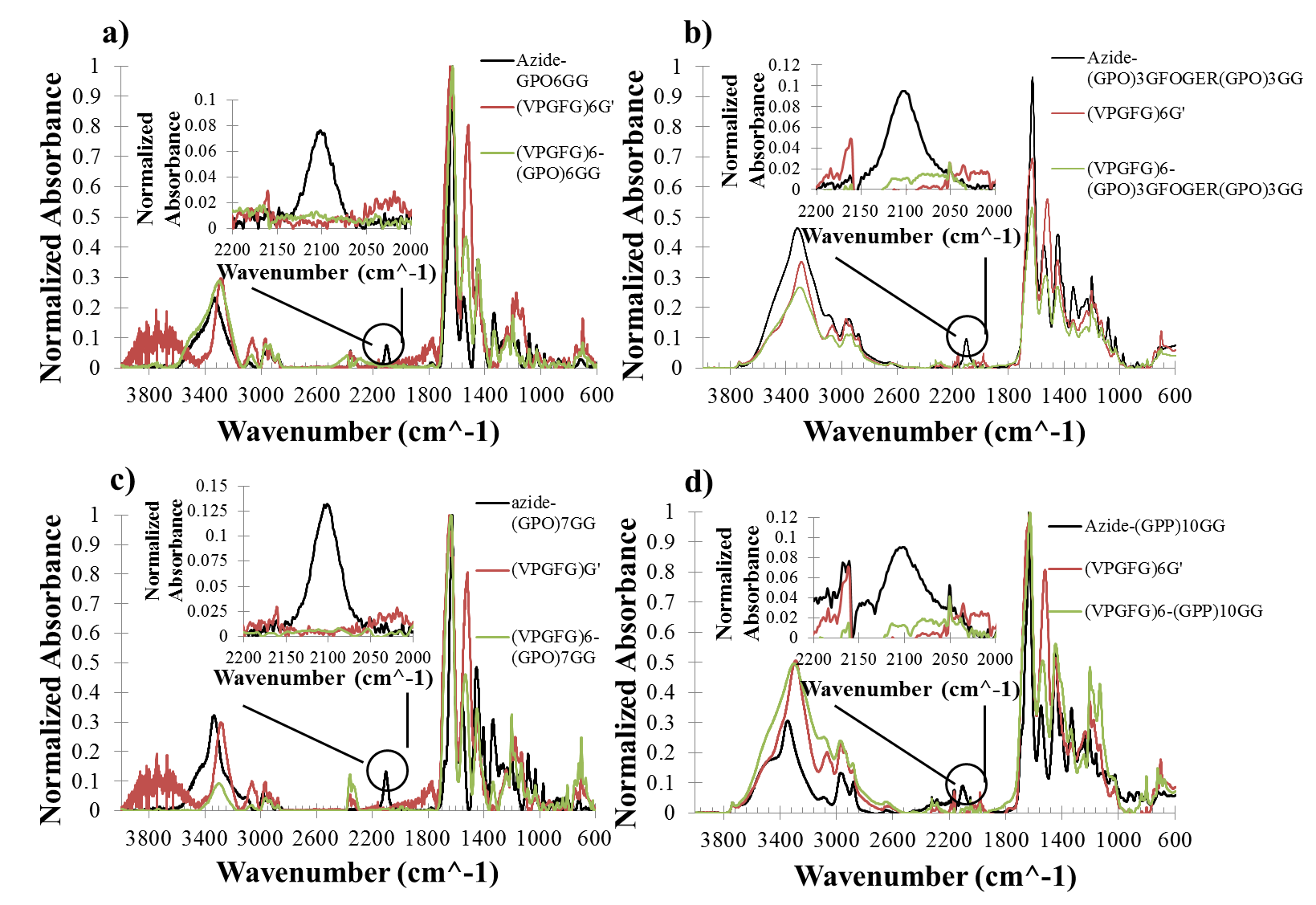


**Figure S12**. Attenuated total reflectance (ATR) Fourier Transform Infrared Spectroscopy (FTIR) of a) (VPGFG)6-alkyne, azide-(GPO)6GG and (VPGFG)6-(GPO)6GG; b) (VPGFG)6-alkyne, azide-(GPO)3GFOGER(GPO)3 GG and (VPGFG)6-(GPO)3GFOGER(GPO)3 GG; c) (VPGFG)6-alkyne, azide-(GPO)7GG and (VPGFG)6-(GPO)7GG; d) (VPGFG)6-alkyne, azide-(GPP)10GG and (VPGFG)6-(GPP)10GG. In each panel the CLP is represented by black lined data, ELP by red lined data, and ELP-CLP conjugate by green lined data. Each panel contains an inset with a closer look at the region of interest (of the azide peak) within the overall FTIR spectra. The presence of the peak at ~2100 cm^-1^ for the CLPs but not for the ELPs or the ELP-CLP conjugates indicates that click chemistry reaction and subsequent HPLC purification was successful. Different batches of (VPGFG)6G’ were used between sets a) / c) and b) / d).


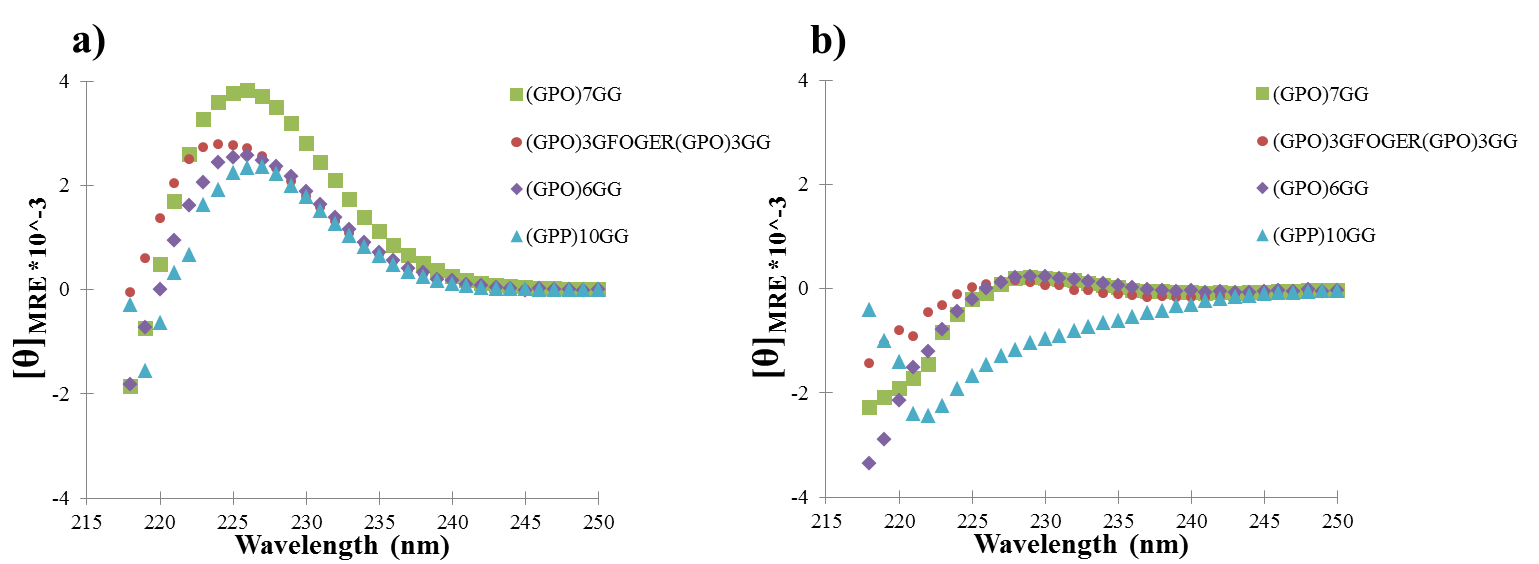


**Figure S13**. Circular dichroism spectroscopy wavelength scans of purified collagen-like peptides azide-(GPO)6GG, azide-(GPO)3GFOGER(GPO)3GG, azide-(GPO)7GG, and azide-(GPP)10GG at: a) 4°C and b) 80°C. Note that the legends of a) and b) have the azide label omitted for brevity. All CLPs have an N-terminal azide functionality. All CLPs were weighed and dissolved in HPLC grade water (pH 6.5) to a concentration of 0.35 mM and were incubated at 4°C overnight prior to CD analysis for a). The scans in b) were taken after the melting curve transitions as well as an additional 0.5 hour incubation time at 80°C. The peaked mean residue ellipticity in a) corresponds to the presence of a structurally intact triple helix for each CLP, while the lack of peaks in b) is representative of a thermally melted state in which the CLPs are no longer triple helices but single stranded.


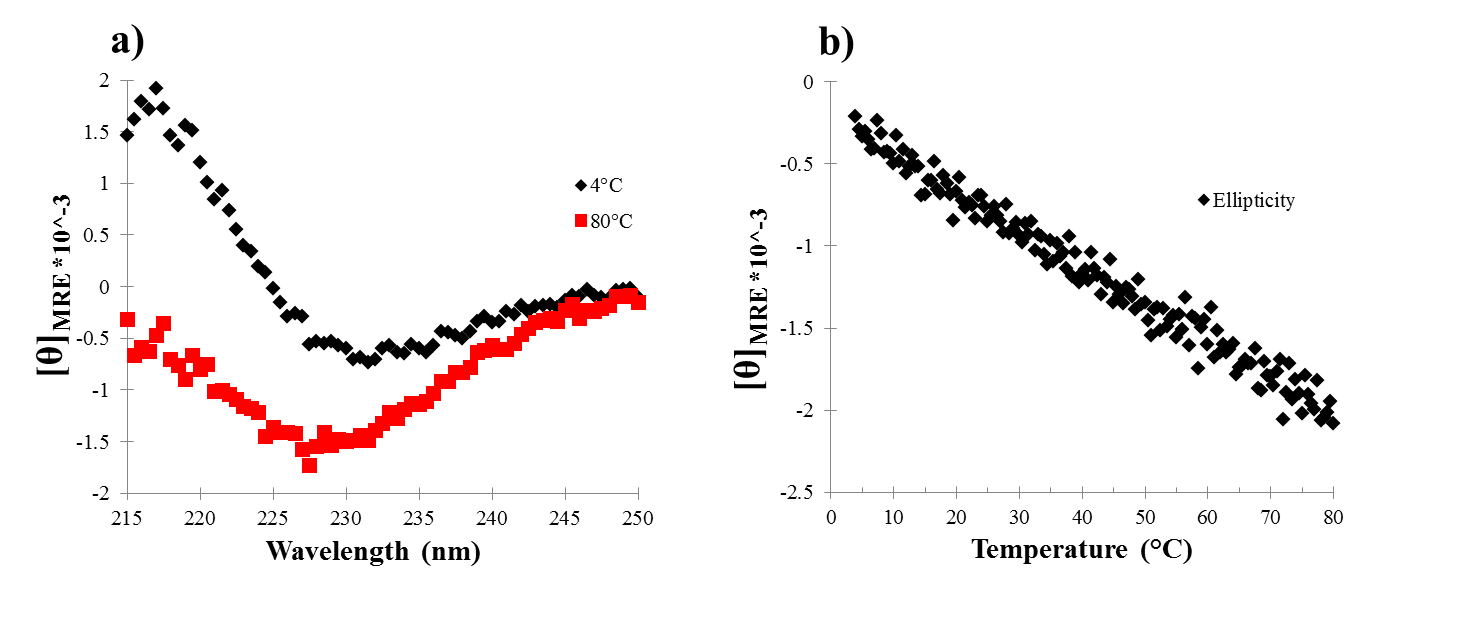


**Figure S14**. Circular dichroism spectroscopy of purified elastin-like peptide (VPGFG)6G’; a) wavelength scans at 4°C (black diamonds) and 80°C (red squares), b) monitoring the 225 nm wavelength ellipticity of the ELP as a function of temperature. The ELP was weighed and dissolved in HPLC grade water (pH 6.5) to a concentration of 0.35 mM. The ELP was incubated at 4°C overnight prior to CD analysis at 4°C in a). The 80°C wavelength scan in a) was taken after the melting curve transition in b) as well as an additional 0.5 hour incubation time at 80°C. The linear decrease of the ELPs ellipticity at 225 nm with respect to increasing temperature is indicative of no transition occurring across the temperature range.^3^


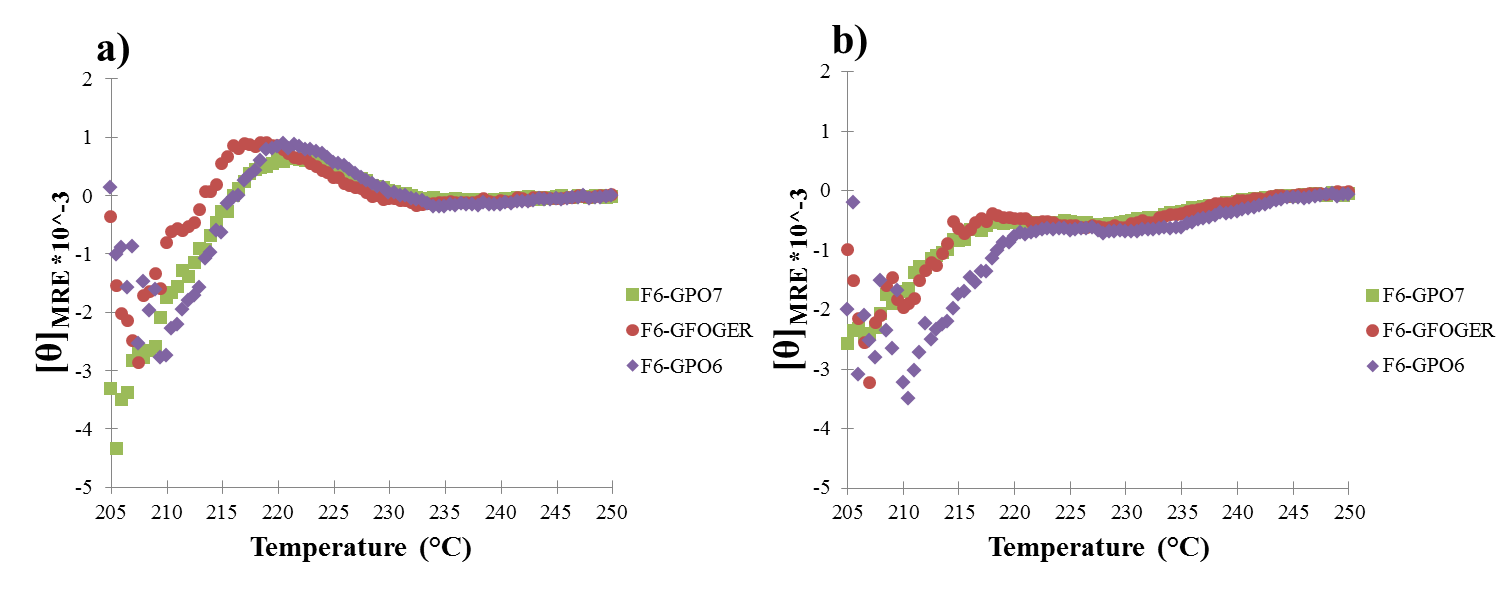


**Figure S15**. Circular dichroism spectroscopy wavelength scans of purified ELP-CLP conjugates (VPGFG)6-(GPO)6GG (F6-GPO6), (VPGFG)6-(GPO)3GFOGER(GPO)3GG (F6-GFOGER), and (VPGFG)6-(GPO)7GG (F6-GPO7) at: a) 4°C and b) 80°C. All conjugates were weighed and dissolved in HPLC grade water (pH 6.5) to a concentration of 0.1 mM and were incubated at 4°C overnight prior to CD analysis. The peaked mean residue ellipticity in a) corresponds to the presence of a structurally intact triple helix for each ELP-CLP conjugate, while the lack of peaks in b) is representative of a thermally melted state in which the ELP-CLPs are no longer in a triple helical state but are single stranded monomeric units. The shift in the maximal peak intensity from 225 nm to 220 nm in a) is most likely attributed to the ELP which begins to have positive ellipticity at 4°C around the 220nm wavelength (see Figure S14a). It should briefly be noted here that the spectra correspond distinctly to triple helices. Though the existence of β-turn secondary structures for ELPs in the collapsed state has been reported before in the literature, we show no conclusive evidence for their presence in these conjugates.


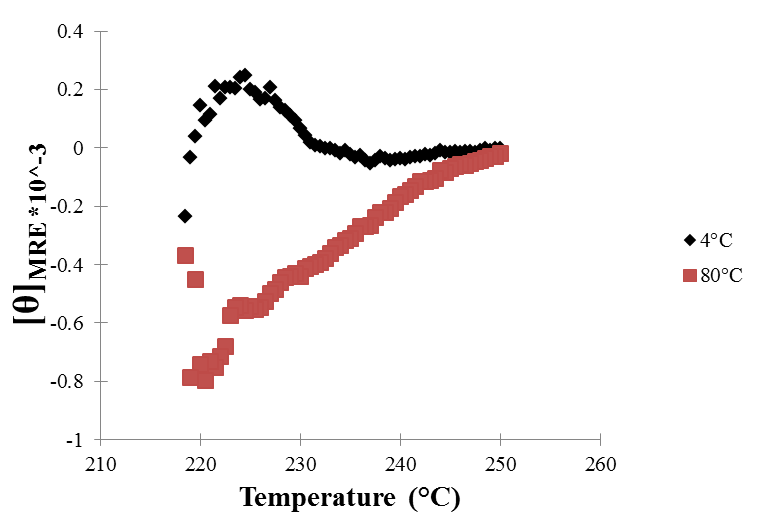


**Figure S16**. Circular dichroism spectroscopy wavelength scans of purified ELP-CLP conjugate (VPGFG)6-(GPP)10GG (F6-GPP10) at 4°C (black diamonds) and 80°C (maroon squares). All conjugates were weighed and mixed in HPLC grade water (pH 6.5) to a concentration of 0.35 mM. Heating to 80°C for 30 minutes was required for conjugate dissolution. The heated sample was then directly transferred to a preheated cuvette and placed in the CD instrument well that was also preheated to 80°C; the 80°C wavelength scan then directly followed cuvette insertion. After the 80°C measurement, the cuvette well was then cooled to 4°C and maintained at that temperature for 30 minutes. This specific procedure was followed to attempt to acquire a positive triple helical signal prior to conjugate precipitation. The data show a weak triple helical signal (positive peak at 225nm) at 4°C and no triple helix at 80°C.


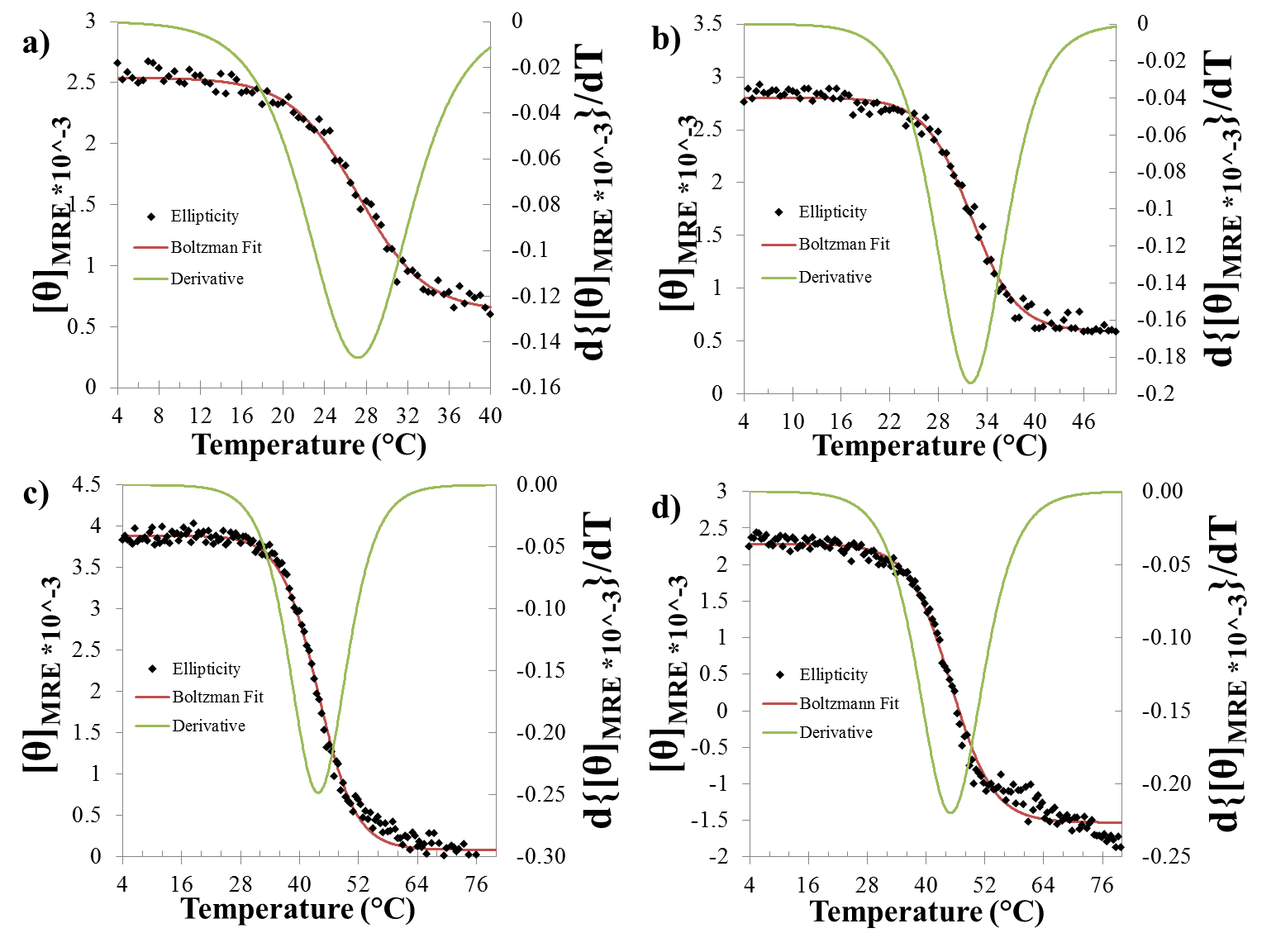


**Figure S17**. Circular dichroism spectroscopy melting temperature determination of purified collagen-like peptides: a) azide-(GPO)6GG, b) azide-(GPO)3GFOGER(GPO)3GG, c) azide-(GPO)7GG and d) azide-(GPP)10GG. All CLPs were weighed and dissolved in HPLC grade water (pH 6.5) to a concentration of 0.35mM. Each melting curve scan is preceded by an overnight incubation at 4°C as well as the 4°C wavelength scan. In each panel the black diamonds represent the mean residue ellipticity, the red line represents a Boltzmann fit of this ellipticity data, and the green curve (and secondary axis) is the first derivative of the Boltzmann fit. The melting temperature is defined as the minima of the first derivative of the Boltzmann fit.


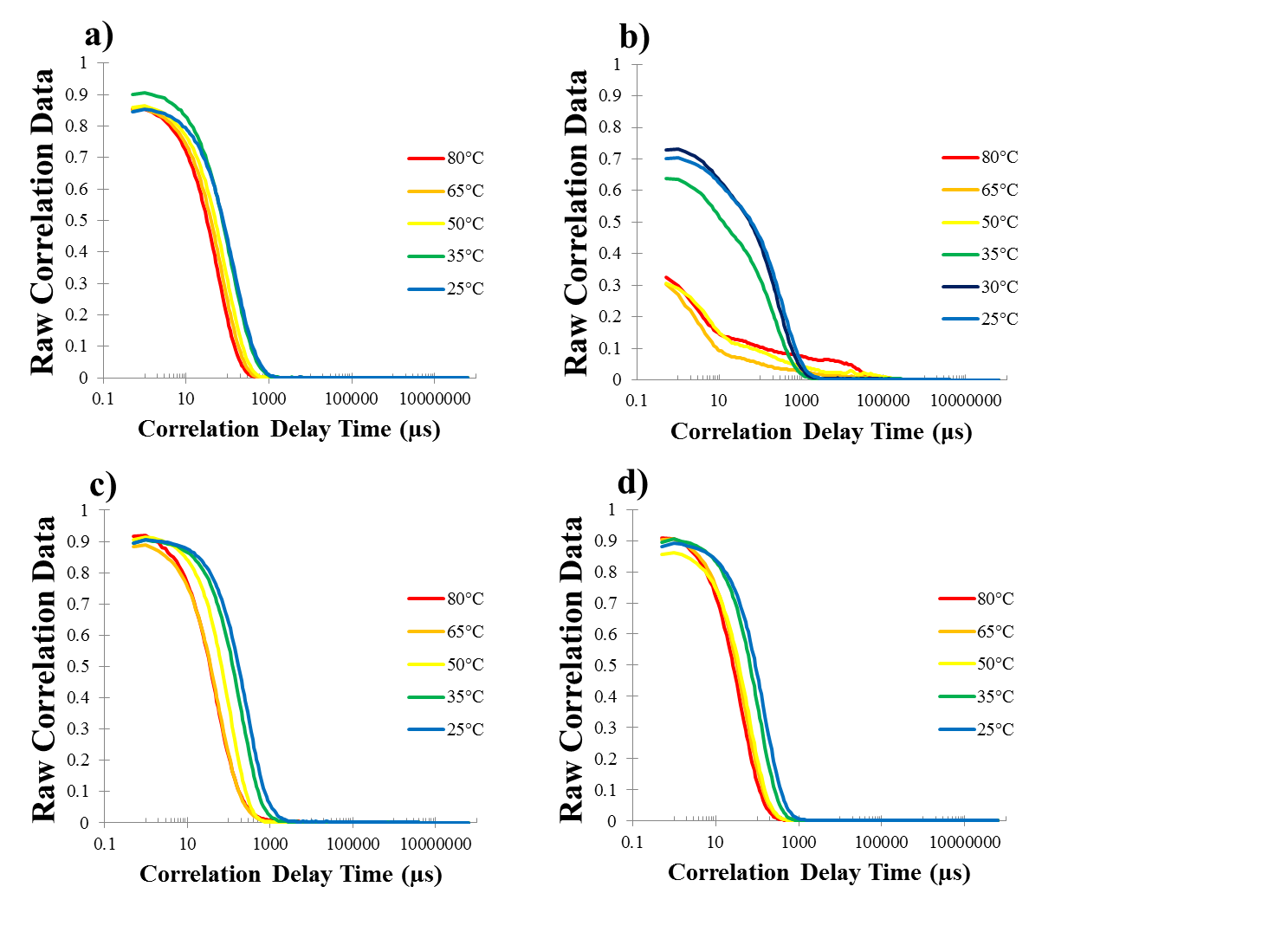


**Figure S18**. Dynamic light scattering correlation decay signals of the conjugates: a) F6-GPO6, b) F6-GFOGER, c) F6-GPO7, and d) F6-GPP10 in HPLC grade water (pH 6.5) at a concentration of 1 mg/mL. For each conjugate (panel), vesicle formation was attempted by starting from 80°C (red line) and cooled sequentially from 65°C (orange line), 50°C (yellow line), 35°C (green line), and 25°C (blue line). The extra temperature of 30°C (purple line) was included for the F6-GFOGER conjugate. Between each cooling step, a five minute equilibration was completed as well as three 200 second measurements, resulting in a total time of each temperature step to be approximately 15 minutes. From the data, only conjugates F6-GPO7 and F6-GPP10 saw a substantial increase in correlation delay time as a function of cooling, indicating the vesicles had formed.

**Table S1**. Number average hydrodynamic diameters of ELP-CLP conjugates as a function of temperature obtained by DLS. Only the F6-GPO7 and F6-GPP10 were observed to have any increases in size during cooling, indicating that only these conjugates could self-assemble. The average diameters and the standard error obtained from measurements of three separate synthetic batches (two for F6-GPP10) are reported.


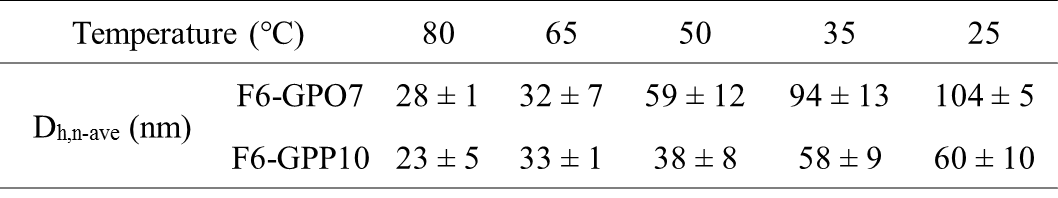


**Table S2**. Summary of the degree of helicity (fraction of folded triple helix) for F6-GPO7, F6-GFOGER, and F6-GPO6 conjugates at their respective T_m_ values. The fraction of folded triple helix was calculated from the Boltzmann fit of each set of melting curve data (Figure 1 in main text) and equation 1.2 above (see section 1.4). The data show that the % of triple helix folded was nearly identical for each conjugate at their respective T_m_ values yet self-assembly was only observed at the T_m­_ for F6-GPO7. This lead us to speculate that the “strength” of the triple helix formation (i.e. the T_m­_) of a conjugate was of greater importance than the increase in local concentration of three ELP chains with regard to whether self-assembly would occur.


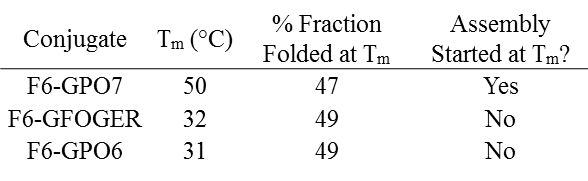


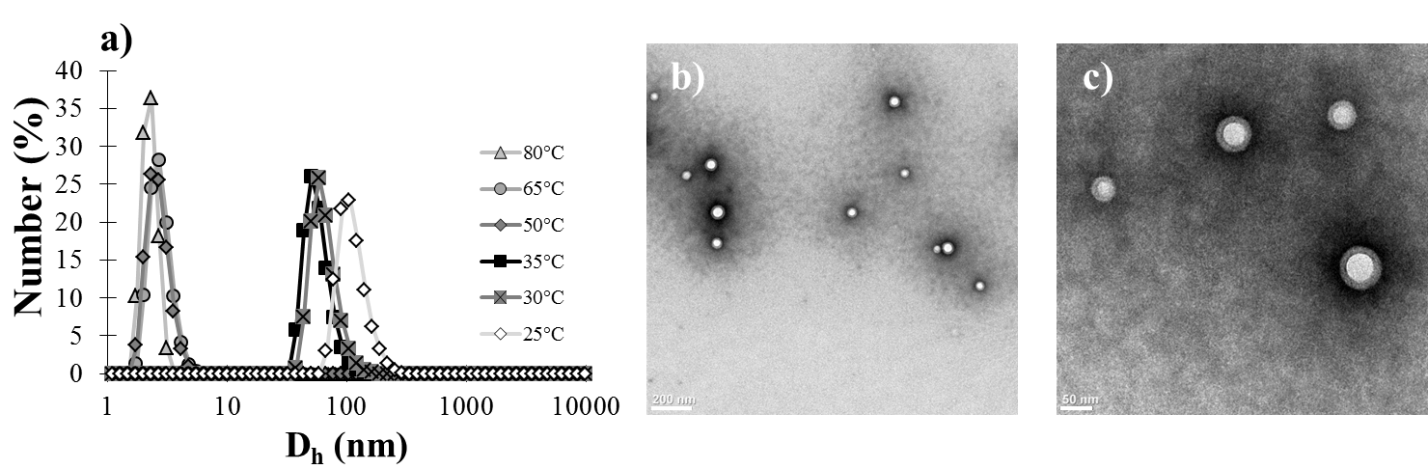


**Figure S19. C**haracterization of F6-GFOGER vesicles in 100 mM NaCl aqueous solution. a) DLS number average D_h_ distribution profiles of F6-GFOGER as function of cooling to determine vesicle formation and size. The F6-GFOGER conjugate was heated to 80°C and subsequently cooled and measured at 80°C (light gray line and triangle), 65°C (gray line and circle), 50°C (gray line and diamond), 35°C (black line and square), 30°C (gray line and x marked gray square), and lastly to 25°C (light gray line and white diamond). b) Transmission electron microscopy at 25°C for determination of F6-GFOGER vesicle morphology; scale bar is 200 nm. c) Second representative transmission electron microscopy image of F6-GFOGER vesicles for closer inspection and sizing of apparent vesicular bilayer; scale bar is 50 nm. The vesicles shown in panels b) and c) were stained with the mutli-step PTA staining procedure (Supplemental Information section 1.5). The diameter of the vesicles and bilayers (measured over multiple images) was determined to be 52 ± 10 nm (n = 43 ) and 13.3 ± 3.3 nm (n = 37), respectively.


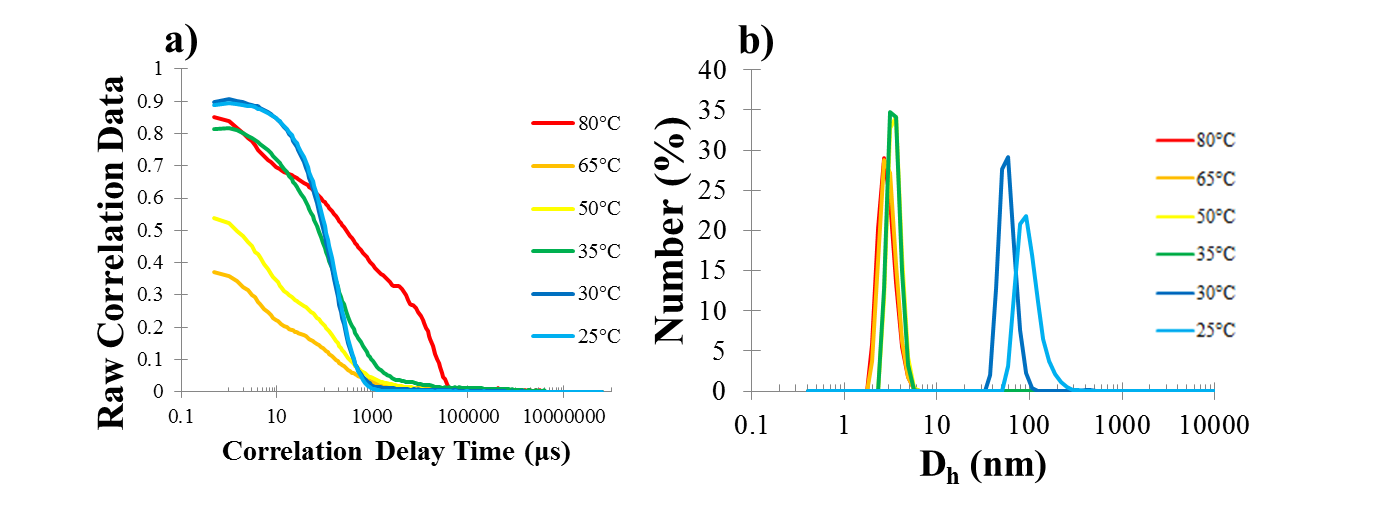


**Figure S20**. DLS analysis of 1 mg/mL F6-GFOGER conjugate vesicle formation in 100 mM NaCl: a) raw autocorrelation decay profiles corresponding to the number average diameter distributions in Figure 19a, b) replicate batch of number average diameter distributions of F6-GFOGER vesicles being formed in 100 mM NaCl solution.


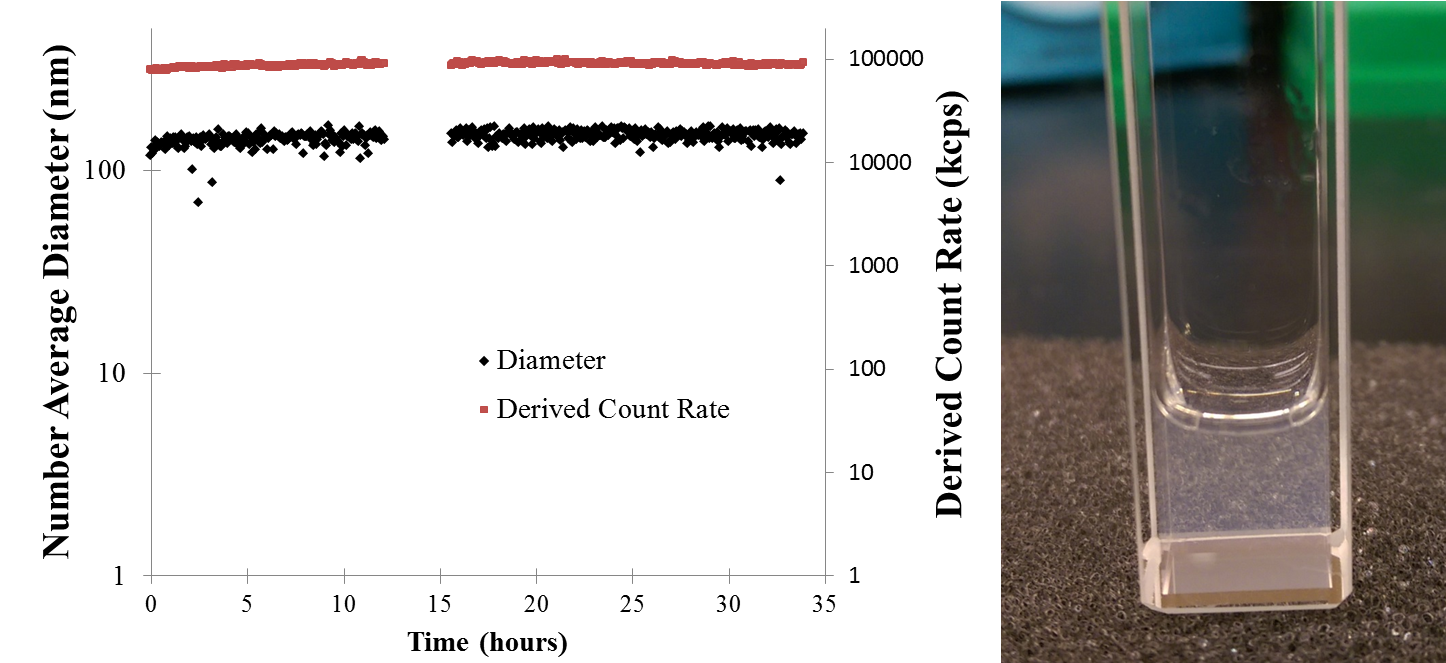


**Figure S21**. Dynamic light scattering of F6-GPO7 vesicles that were monitored at 25°C for over 24 hours along with a corresponding image of the vesicles (in the cuvette) that were monitored during the experiment. The DLS data show that the vesicle scattering (in the form of the attenuation corrected photon count rate) and the hydrodynamic diameter did not change over the course of the 34 hour experiment. This result indicated that the vesicles were colloidally stable and therefore ideal for drug delivery applications. The image of the vesicles further confirms the colloidally stable vesicles by the lack of settled aggregates and large degree of a cloudy dispersion present in the cuvette. The gap in the DLS data was intentional so that another user could use the instrument.


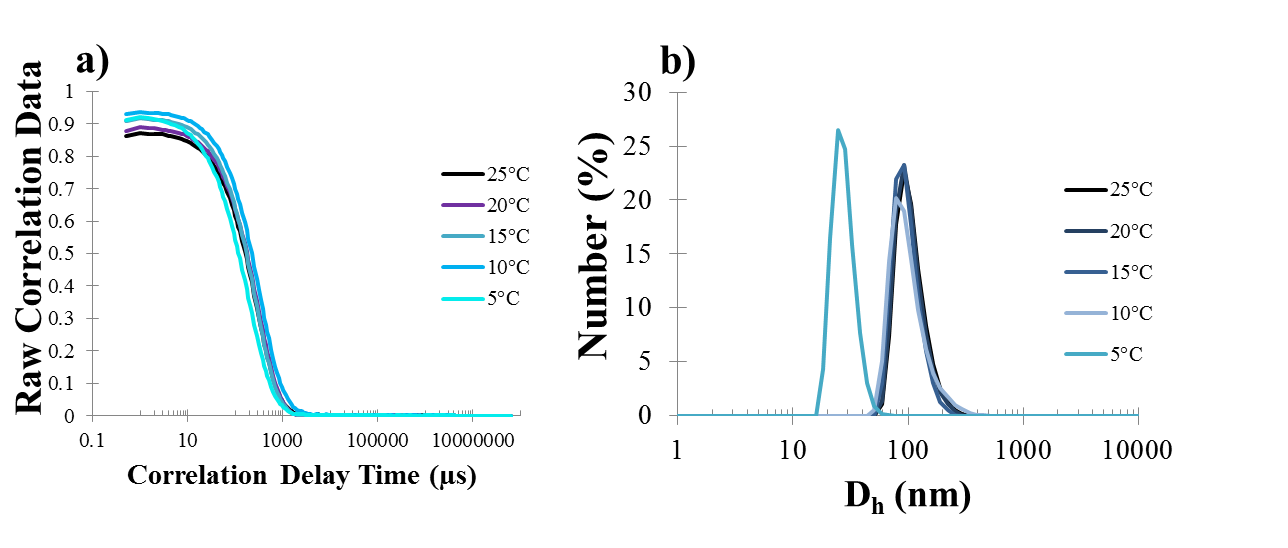


**Figure S22**. Dynamic light scattering of pre-formed F6-GPO7 vesicles as a function of cooling: a) raw autocorrelation decay profiles corresponding to the data in Figure 3b) in the text, and b) number diameter distribution data for a second replicate batch of F6-GPO7 vesicles. The data show a) the reduction in delay time of the autocorrelation function between the temperatures of 10°C and 5°C and b) the significant drop in the number diameter distribution between these two temperatures as well.


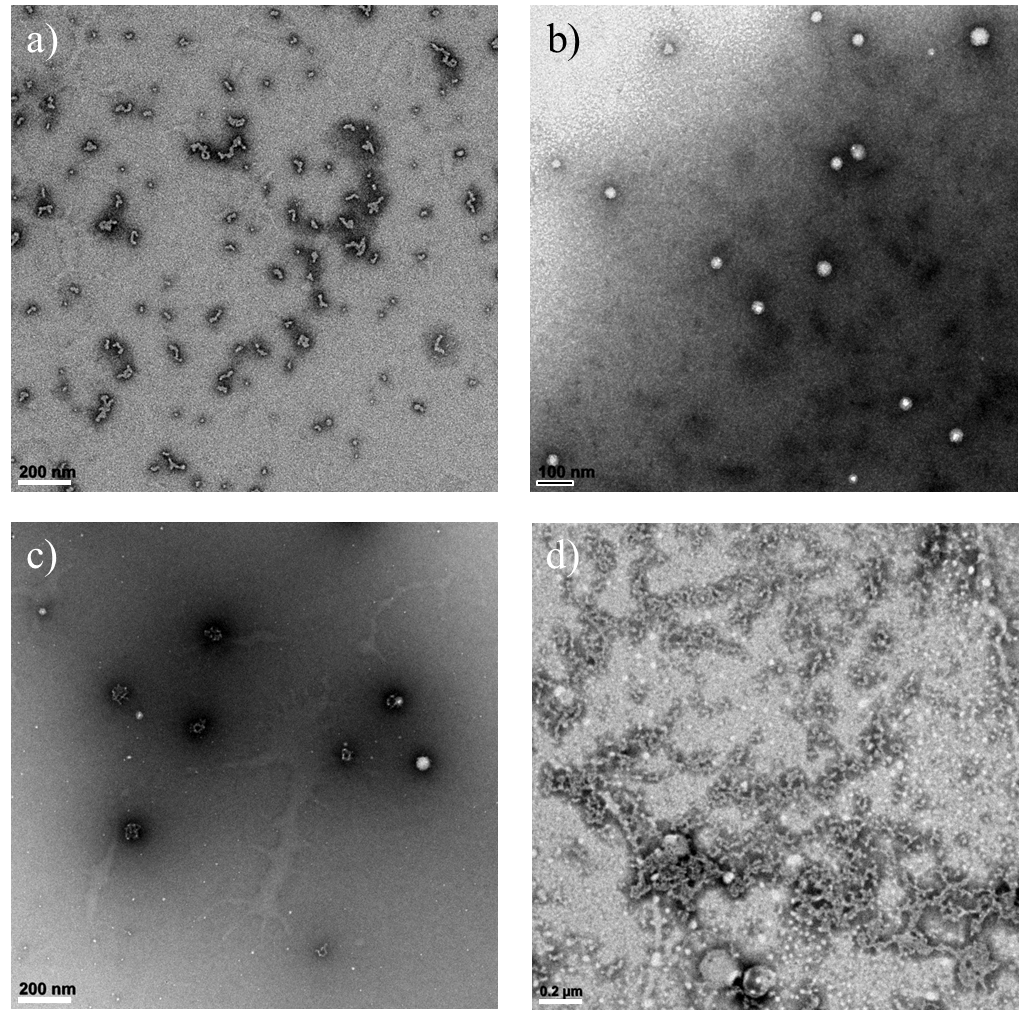


**Figure S23**. Replicate transmission electron microscopy images of dually thermoresponsive F6-GPO7 vesicles that were prepared at the temperatures: a) 4°C, b) 25°C, c) 50°C and d) 80°C. All vesicles were stained with 1wt% PTA following the methods described in section 2.6 of the main text. The scale bars of a), c), and d) corresponds to 200 nm, while the scale bar of b) corresponds to 100 nm (different scale bar formatting). The images highlight the dual thermoresponsive nature of the F6-GPO7 vesicles.


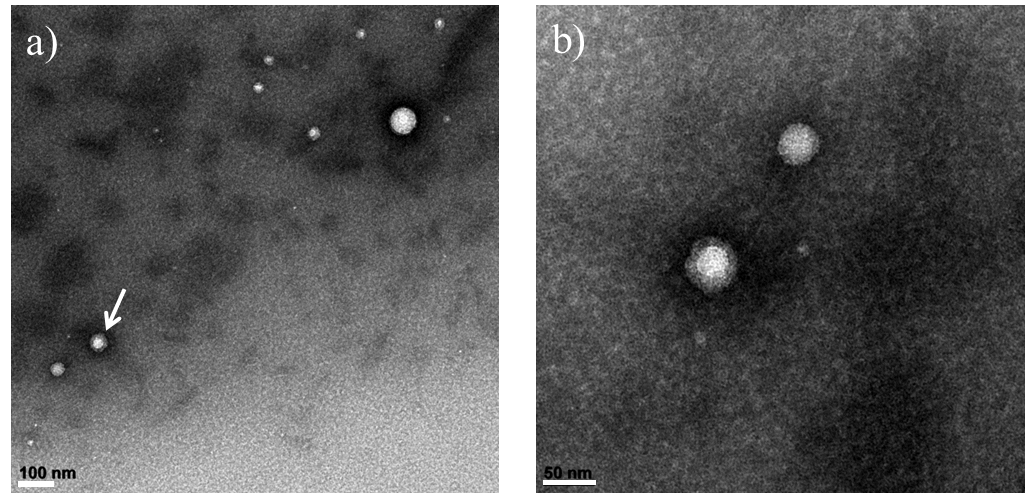


**Figure S24**. Representative TEM images of F6-GPO7 vesicles that were prepared at 25°C using the 3x PTA staining technique (see section 1.4 above). Scale bar in a) is 100 nm and the scale bar in b) is 50 nm. The white arrow in a) distinguishes a nanovesicle with an apparent bilayer structure. Note that b) is a magnified image of the vesicle distinguished by the white arrow in a). The length of the bilayer is 11.5 ± 2 nm (n = 32).


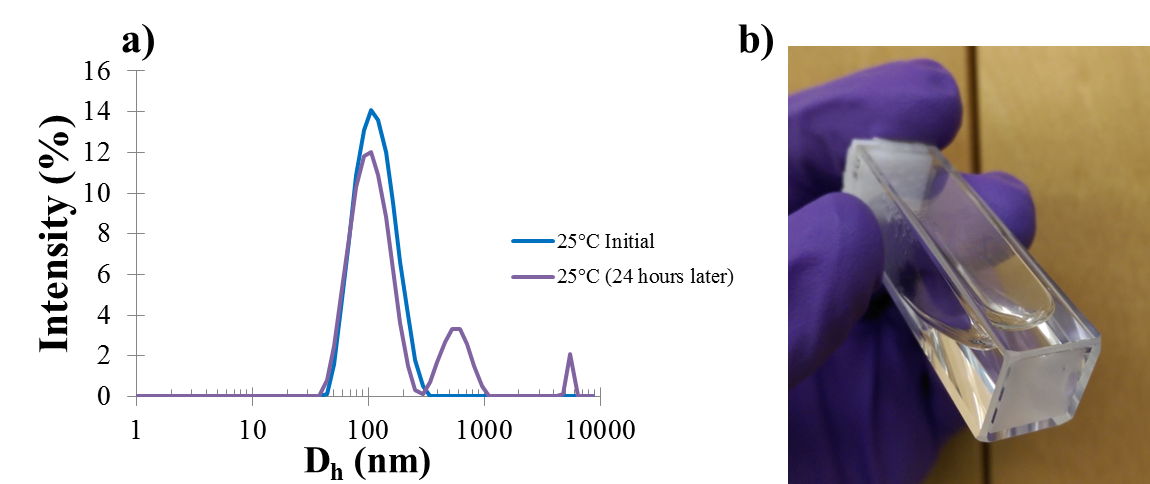


**Figure S25**. F6-GPP10 aggregation observations: a) dynamic light scattering intensity weighted particles diameter distributions of F6-GPP10 particles / aggregates at an initial time point and 24 hours later, both at 25°C, b) image of the settled aggregates inside the cuvette near the 24 hour time point in a). The data show that the F6-GPP10 particles aggregate over time.


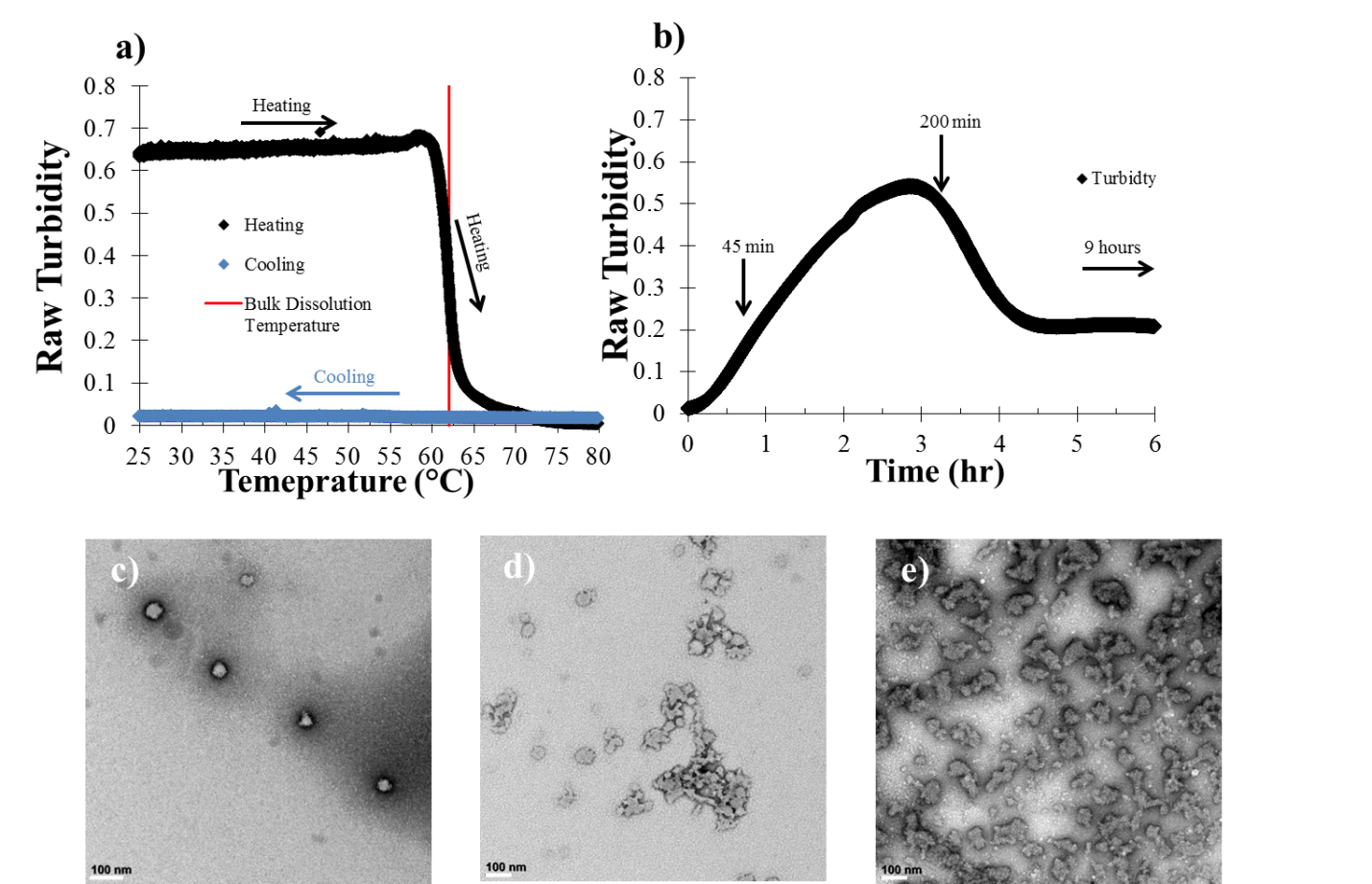


**Figure S26. C**haracterization of F6-GPP10 aggregates. a) Turbidity measurements of F6-GPP10 aggregates (preformed 24 hours prior to measurement (Figure S25)) during heating and stirring (2.5°C/min and 400rpm, marked by black diamond data points) followed by cooling (2.5°C/min, marked by blue diamond data points) with no stirring. A red line marks the point in which dissolution of the aggregates reaches 50% of maximal turbidity and arrows show the directionality in which data was recorded, with heating occurring first. b) Continuous turbidity monitoring following the cooling measurement in a) show the process of aggregate formation, settling, and equilibration over the course of 6 hours with no stirring. Arrows and labels mark time points in which aliquots of the solution were removed for TEM analysis. c),d) and e) Representative TEM images of the 45, 200, and 540 minute time points that were aliquoted from b) and depicts discrete non-spherical particles, particle clumping/aggregation and large aggregates respectively; all scale bars are 100nm.

**References:**

[1] Presolski, S. I.; Hong, V. P.; Finn, M. G., Copper-Catalyzed Azide-Alkyne Click Chemistry for Bioconjugation. *Current protocols in chemical biology* **2011,** *3*(4), 153-162.

[2] Lambert, J.B.; Shurvell, H.F.; Lightner, D.A.; Cooks, R.G., Electronic Absorption and Chiroptical Spectroscopy. In Organic Structural Spectroscopy; Prentice-Hall: New Jersey, **1998**.

[3] Greenfield, N. J., Analysis of the kinetics of folding of proteins and peptides using circular dichroism. *Nature Protocols* **2006,** *1* (6), 2891-2899.

[4] Engel, J.; Bachinger, H. P., Structure, stability and folding of the collagen triple helix. In *Collagen: Primer in Structure, Processing and Assembly*, Brinckmann, J.; Notbohm, H.; Muller, P. K., Eds. Springer-Verlag Berlin: Berlin, **2005**; Vol. 247, pp 7-33.

[5] Urry, D. W., Physical chemistry of biological free energy transduction as demonstrated by elastic protein-based polymers. *Journal of Physical Chemistry B* **1997,** *101* (51), 11007-11028.

[6] Reguera, J.; Urry, D. W.; Parker, T. M.; McPherson, D. T.; Rodriguez-Cabello, J. C., Effect of NaCl on the exothermic and endothermic components of the inverse temperature transition of a model elastin-like polymer. *Biomacromolecules* **2007,** *8* (2), 354-358.

[7] Urry, D. W., MOLECULAR MACHINES - HOW MOTION AND OTHER FUNCTIONS OF LIVING ORGANISMS CAN RESULT FROM REVERSIBLE CHEMICAL-CHANGES. *Angewandte Chemie-International Edition in English* **1993,** *32* (6), 819-841.

[8] Shoulders, M. D.; Raines, R. T., Collagen Structure and Stability. In *Annual Review of Biochemistry*, Annual Reviews: Palo Alto, **2009**; Vol. 78, pp 929-958.

[9] Brodsky, B.; Ramshaw, J. A. M., The collagen triple-helix structure. *Matrix Biology* **1997,** *15* (8-9), 545-554.

[10] Rodriguez-Cabello, J. C.; Prieto, S.; Reguera, J.; Arias, F. J.; Ribeiro, A., Biofunctional design of elastin-like polymers for advanced applications in nanobiotechnology. *Journal of Biomaterials Science-Polymer Edition* **2007,** *18* (3), 269-286.

[11] Urry, D. W., PROTEIN ELASTICITY BASED ON CONFORMATIONS OF SEQUENTIAL POLYPEPTIDES - THE BIOLOGICAL ELASTIC FIBER. *Journal of Protein Chemistry* **1984,** *3* (5-6), 403-436.

[12] Tadros, T. F., Interfacial Phenomena and Colloid Stability: Basic Principles, Vol 1. *Interfacial Phenomena and Colloid Stability: Basic Principles, Vol 1* **2015**, 1-342.

[13] Mai, Y. Y.; Eisenberg, A., Self-assembly of block copolymers. *Chemical Society Reviews* **2012,** *41* (18), 5969-5985.

[14] Israelachvili, J. N., Intermolecular and Surface Forces, 3rd Edition. *Intermolecular and Surface Forces, 3rd Edition* **2011**, 1-674.
